# The *in Vitro* Inhibitory Effect of Ectromelia Virus Infection on Innate and Adaptive Immune Properties of GM-CSF-Derived Bone Marrow Cells Is Mouse Strain-Independent

**DOI:** 10.3389/fmicb.2017.02539

**Published:** 2017-12-19

**Authors:** Lidia Szulc-Dąbrowska, Justyna Struzik, Joanna Cymerys, Anna Winnicka, Zuzanna Nowak, Felix N. Toka, Małgorzata Gieryńska

**Affiliations:** ^1^Department of Preclinical Sciences, Faculty of Veterinary Medicine, Warsaw University of Life Sciences, Warsaw, Poland; ^2^Department of Pathology and Veterinary Diagnostics, Faculty of Veterinary Medicine, Warsaw University of Life Sciences, Warsaw, Poland; ^3^Department of Genetics and Animal Breeding, Faculty of Animal Sciences, Warsaw University of Life Sciences, Warsaw, Poland; ^4^Department of Biomedical Sciences, Ross University School of Veterinary Medicine, Basseterre, Saint Kitts and Nevis

**Keywords:** ectromelia virus, conventional dendritic cells, Th polarization, immunosuppression, viral evasion strategies

## Abstract

Ectromelia virus (ECTV) belongs to the *Orthopoxvirus* genus of the *Poxviridae* family and is a natural pathogen of mice. Certain strains of mice are highly susceptible to ECTV infection and develop mousepox, a lethal disease similar to smallpox of humans caused by variola virus. Currently, the mousepox model is one of the available small animal models for investigating pathogenesis of generalized viral infections. Resistance and susceptibility to ECTV infection in mice are controlled by many genetic factors and are associated with multiple mechanisms of immune response, including preferential polarization of T helper (Th) immune response toward Th1 (protective) or Th2 (non-protective) profile. We hypothesized that viral-induced inhibitory effects on immune properties of conventional dendritic cells (cDCs) are more pronounced in ECTV-susceptible than in resistant mouse strains. To this extent, we confronted the cDCs from resistant (C57BL/6) and susceptible (BALB/c) mice with ECTV, regarding their reactivity and potential to drive T cell responses following infection. Our results showed that *in vitro* infection of granulocyte-macrophage colony-stimulating factor-derived bone marrow cells (GM-BM—comprised of cDCs and macrophages) from C57BL/6 and BALB/c mice similarly down-regulated multiple genes engaged in DC innate and adaptive immune functions, including antigen uptake, processing and presentation, chemokines and cytokines synthesis, and signal transduction. On the contrary, ECTV infection up-regulated *Il10* in GM-BM derived from both strains of mice. Moreover, ECTV similarly inhibited surface expression of major histocompatibility complex and costimulatory molecules on GM-BM, explaining the inability of the cells to attain full maturation after Toll-like receptor (TLR)4 agonist treatment. Additionally, cells from both strains of mice failed to produce cytokines and chemokines engaged in T cell priming and Th1/Th2 polarization after TLR4 stimulation. These data strongly suggest that *in vitro* modulation of GM-BM innate and adaptive immune functions by ECTV occurs irrespective of whether the mouse strain is susceptible or resistant to infection. Moreover, ECTV limits the GM-BM (including cDCs) capacity to stimulate protective Th1 immune response. We cannot exclude that this may be an important factor in the generation of non-protective Th2 immune response in susceptible BALB/c mice *in vivo*.

## Introduction

The poxviruses are large DNA viruses that are undoubtedly masters of immune evasion, and have evolved to modulate and inhibit the host immune and inflammatory responses. The poxvirus genome encodes multiple classes of immunomodulatory proteins that act either intracellularly (virostealth and virotransducers) or extracellularly (viromimetics: virokines and viroceptors) and antagonize or compete with molecules critically involved in the host antiviral response. In fact, due to diversity of these genes, a single immunomodulatory protein that is shared by all poxviruses has not been identified yet. Moreover, a unique repertoire of immunomodulatory proteins encoded by each virus species allows it to successfully evade the immune response and survive in its natural host (Stanford et al., 2007). Highly host-specific survival strategy is employed especially by members of the *Orthopoxvirus* genus that exhibit a narrow host range and co-evolved with their natural host, e.g., variola virus (VARV, the causative agent of smallpox) in human and ectromelia virus (ECTV, the causative agent of mousepox) in mice. Meantime, other orthopoxviruses, such as vaccinia (VACV), monkeypox (MPXV), and cowpox (CPXV) viruses, which have a broad host range, are able to infect many different mammalian species and may contribute to the unpredictable outcome of infection in a new host species, e.g., MPXV in humans (McCollum and Damon, 2014). Therefore, a better understanding of the immunomodulatory mechanisms used by orthopoxviruses in their natural hosts is especially important for a full knowledge of their immune evasion strategies employed to control the host immune system.

The mousepox model is an excellent small animal model to study pathogenesis of smallpox, a disease that, despite being eradicated from the globe, now represents one of the most dangerous bioterrorism threats to human society. Smallpox is considered by Centers for Disease Control and Prevention (CDC) in Atlanta as a category A bioterrorism agent due to its easy dissemination, transmission from person to person, and high mortality rates (Riedel, 2005). ECTV shares with VARV several common properties, including: narrow host range and co-evolution with the natural host, high infectivity at low dose, and viral transmission and replication. Moreover, both viruses cause severe diseases with similar pathogenesis, aspects of pathology and immune response, and high mortality rates (Stanford et al., 2007). Therefore, mousepox model is extensively used to study basic questions in immune response regulation during generalized viral infections to eventually develop new prophylactic and therapeutic treatments against orthopoxviruses (Parker et al., 2010).

Within inbred strains of mice there is a genetically determined resistance to severe mousepox. C57BL/6 [H-2^b^] mice are resistant to the lethal form of disease, whereas BALB/c [H-2^d^] mice are fully susceptible to ECTV infection and usually succumb to disease between 7 and 9 days post-footpad infection. Genetic resistance is controlled by at least four autosomal dominant genes called *rmp* (resistance to mousepox), which are involved in regulation of some aspects of innate immunity (Brownstein and Gras, 1995). Additionally, during the infection resistant and susceptible strains of mice develop different types of the T helper (Th) cytokine immune responses: C57BL/6 mice generate a protective Th1 immune response accompanied by strong cytotoxic T lymphocyte (CTL) activity, whereas BALB/c mice generate a non-protective Th2 immune response, which is associated with a weak/absent CTL activity (Chaudhri et al., 2004).

The central role in driving T cell responses is played by dendritic cells (DCs), the most potent antigen presenting cells (APCs). Depending on lineage, maturation stage, and activation status, DCs release different polarizing signals, the most important of which are cytokines and chemokines that selectively promote the generation of Th1, Th2, Th17, or regulatory T cells (Tregs) (Kaiko et al., 2008). Immature DCs, characterized by low expression of antigen presenting [major histocompatibility complex (MHC) I, MHC II, CD1d] and costimulatory (CD80, CD86, and CD40) molecules induce anergy in antigen-specific naïve T cells or generate Foxp3^+^ induced Tregs in the presence of transforming growth factor (TGF)-β. Additionally, immature or semi-mature DCs are able to induce regulatory Foxp3^-^ IL-10^+^ Tr1 (type 1 regulatory T) cells. Th2-polarizing DCs have semi-mature state associated with increased expression of antigen presenting and costimulatory molecules, and inability to secrete polarizing cytokines, such as IL-12p70 (Th1) or IL-6 and IL-23 (Th17). Moreover, IL-10 produced by DCs has been associated with propagation of Th2 immunity, however, this cytokine preferentially blocks DC maturation and induces anergy or Tr1 cells. Fully matured DCs, upon lipopolysaccharide (LPS) or CpG oligonucleotide treatment, produce IL-12p70 and possess strong capacity to polarize T cells toward Th1 profile (Lutz, 2016).

As masters of immune evasion, orthopoxviruses are able to control DC functions important for induction of an antiviral immune response. In general, they inhibit numerous functions of these cells, including antigen uptake and presentation, maturation, pro-inflammatory response, and capacity to activate T cells (Engelmayer et al., 1999; Jenne et al., 2000; Hansen et al., 2011; Szulc-Dąbrowska et al., 2017). However, ECTV, unlike VACV and CPXV, can productively infect conventional DCs (cDCs) *in vitro* (Szulc-Dąbrowska et al., 2017) and *in vivo* (Sei et al., 2015), what indicates strong adaptation capacity of ECTV to the natural host immune cells. It has been shown that cDCs derived from resistant C57BL/6 and susceptible BALB/c mouse strains may differentially react during viral (Pejawar et al., 2005) and bacterial (Jiang et al., 2010) infections *in vitro*. Upon infection, cDCs from C57BL/6 mice underwent higher functional maturation and/or were able to stimulate more potent CD8^+^ T cell response than cells from BALB/c mice (Pejawar et al., 2005; Jiang et al., 2010).

We therefore confronted the cDCs from resistant (C57BL/6) and susceptible (BALB/c) mice regarding inter-strain differences in reactivity and potential to drive T cell responses following *in vitro* infection with ECTV. Our results showed that ECTV similarly affects innate and adaptive immune functions of granulocyte-macrophage colony-stimulating factor (GM-CSF)-derived bone marrow cells (GM-BM) obtained from both mouse strains. GM-BM infected with ECTV exhibited a profound down-regulation in expression of many genes involved in maturation and activation of DCs, with the exception of *Il10*, which was up-regulated in a strain independent manner. Moreover, ECTV impaired production of chemokines and cytokines engaged in regulation of Th1 and Th2 immune responses, as well as decreased maturation marker expression on the surface of C57BL/6 and BALB/c GM-BM. Collectively, our data suggest that ECTV-infected GM-BM have strong potential to silence the Th immune response, independently of their genetic background.

## Materials and Methods

### Animals

Male C57BL/6 (H-2^b^) and BALB/c (H-2^d^) mice (8–12 weeks old) were purchased from the animal facility at Maria Sklodowska-Curie Memorial Cancer Centre and Institute of Oncology in Warsaw, Poland. After arrival, animals were kept for 7 days for acclimatization in the animal facility at the Faculty of Veterinary Medicine under controlled temperature and humidity with free access to food and water. Experimental procedures on animals were approved by the 3rd Ethical Committee for Animal Experimentation at Warsaw University of Life Sciences—SGGW (permission no. 34/2012) and were performed in accordance with institutional Guidelines for Care and Use of Laboratory Animals.

### Virus

The Moscow strain of ECTV (ATCC, VR-1374) was obtained from American Type Culture Collection (Manassas, VA, United States). The virus was propagated on African green monkey kidney (Vero) cells (ATCC, CCL-81) maintained in DMEM high glucose (HyClone, Logan, UT, United States) supplemented with 5% fetal bovine serum (FBS; HyClone) and 1% antibiotic–antimycotic solution (100 U/ml penicillin, 100 μg/ml streptomycin, and 0.25 μg/ml amphotericin B; Sigma–Aldrich, St. Louis, MO, United States). The virus stock was purified by sucrose cushion centrifugation. Briefly, virus suspension was layered onto 36% sucrose in 1 mM Tris pH 9.0 and centrifuged at 30,000 × *g* for 60 min at 4°C. After purification, virus stock infectivity was determined by plaque formation assay (PFU/ml) on Vero cell monolayer. Inactivation of the virus was performed by a 30 min exposure to UV radiation (120 W, 320 nm) at a working distance of 12 cm from the UV lamp. The absence of plaque formation in the Vero cell monolayer indicated complete inactivation of the virus.

### Generation and Enrichment of GM-BM

The protocol used to generate GM-BM was similar to that used by Lutz et al. (1999). Bone marrow was flushed with cold RPMI-1640 medium from femurs and tibias of mice and erythrocytes were removed using ammonium chloride buffer. Cells were then washed and plated at 1 × 10^6^/well in a six-well plate in RPMI-1640 medium containing 10% heat-inactivated FBS, 1% antibiotic solution (100 U/ml penicillin and 100 mg/ml streptomycin; Sigma–Aldrich), 50 μM 2-mercaptoethanol (Sigma–Aldrich), and 20 ng/ml recombinant mouse (rm) GM-CSF (R&D Systems, Minneapolis, MN, United States). Fresh medium with 20 ng/ml rmGM-CSF was added at day 3 of culture and then was partially replaced every 2 days. On day 8 post-culture, cDCs were enriched using MACS CD11c^+^ labeled magnetic beads (Miltenyi Biotec, Auburn, CA, United States). After MACS separation GM-BM cultures were evaluated for surface expression of cellular markers using the following monoclonal antibodies (mAbs): anti-mouse CD11c-BV421 (N418, Armenian Hamster IgG2; BioLegend, San Diego, CA, United States), anti-CD11b-BV605 (M1/70, rat IgG2b), anti-I-A/I-E-BV711 (M5/114.15.2, rat IgG2b; both from BD Biosciences, San Jose, CA, United States) and anti-CD205-PE-Cy7 (NLDC-145, rat IgG2a; BioLegend) (**Supplementary Figure S1**).

### GM-BM Infection and Treatment

MACS-separated CD11c^+^ cells (purity ≥95%) were infected with live ECTV at multiplicity of infection (MOI) of 1. In some experiments, cells were exposed to UV-inactivated ECTV (uvi-ECTV) at MOI of 1 (before inactivation). As a control, non-infected cells were cultured in parallel in complete RPMI-1640 medium. Additionally, non-, ECTV-, and/or uvi-ECTV-exposed cells were left untreated or were treated for 24 h with 1 μg/ml LPS (*Escherichia coli* 0111:B4; Sigma–Aldrich), which is a Toll-like receptor (TLR)4 agonist used as a positive control for fully matured cells.

### Plaque Assay

Vero cells cultured on 24-well plates were treated with 10-fold serial dilutions of ECTV stocks obtained from C57BL/6 and BALB/c GM-BM at 4, 12, and 24 hpi. After 5 days, plaques were counted under Olympus IX71 inverted microscope. After counting, Vero cell monolayers were stained with 0.3% crystal violet and air dried.

### Measurement of Apoptosis

The apoptotic rate of GM-BM was measured using the FITC Annexin V Apoptosis Detection Kit I (BD Biosciences), according to the manufacturer’s protocol. Briefly, cells were washed twice with cold phosphate-buffered saline (Sigma–Aldrich) and resuspended in 1× binding buffer at a concentration of 1 × 10^6^ cells/ml. Then, 100 μl of the solution (containing 1 × 10^5^ cells) was stained with 5 μl Annexin V-FITC and 5 μl propidium iodide (PI) and incubated for 15 min at room temperature in the dark. Finally, cells were resuspended in 400 μl of 1× binding buffer and analyzed immediately by flow cytometry. Viable cells (no measurable apoptosis) are Annexin V-FITC and PI negative, early apoptotic cells (membrane integrity is present) are Annexin V-FITC positive and PI negative, whereas late apoptotic cells (end stage apoptosis and death) are Annexin V-FITC and PI positive.

### Real-Time Reverse Transcription Polymerase Chain Reaction (RT-PCR)

The expression of selected genes involved in innate and adaptive immune functions of GM-BM was evaluated using real-time reverse transcription polymerase chain reaction (RT-PCR), as previously described (Dolega et al., 2017; Szulc-Dąbrowska et al., 2017) with minor modifications. Briefly, RNA was extracted from 1 × 10^6^ mock- or ECTV-infected GM-BM, untreated or treated with LPS for 24 h using Qiagen RNeasy Mini Kit (Qiagen, Inc., Valencia, CA, United States), as recommended by the manufacturer. Additionally, on-column DNase I digestion of genomic DNA was performed using RNase-Free DNase Set (Qiagen). The RNA concentration and purity was assessed using the Take-3 system on Epoch BioTek spectrophotometer and analyzed in Gen5 software (BioTek Instruments, Inc., Winooski, VT, United States). Total RNA (1 μg) was applied for cDNA synthesis using the RT^2^ First Strand Kit (Qiagen) according to the manufacturer’s protocol. Before RT, genomic DNA was additionally removed by incubation in GE2 buffer for 5 min at 42°C. For analysis of selected genes involved in regulation of innate and adaptive immune properties of GM-BM the Mouse Dendritic and Antigen Presenting RT^2^ Profiler PCR Array (Qiagen) was used according to the recommendation of the manufacturer. Briefly, 550 ng cDNA was mixed with RT^2^ SYBR Green Mastermix (Qiagen) and aliquoted into the 96-well RT^2^ Profiler PCR array plate, containing lyophilized RT^2^ qPCR primers for a set of 84 related genes, five housekeeping genes (*Actb, B2m, Gapdh, Gusb*, and *Hsp90ab1*), three Reverse Transcription Controls (RTC), three Positive PCR Controls (PPC), and one Mouse Genomic DNA Contamination (MGDC) control (**Supplementary Table S1**). Amplification was performed in ABI 7500 thermocycler (Life Technologies, Carlsbad, CA, United States) at 95°C for 10 min, 40 cycles of 95°C for 15 s and 60°C for 1 min. Fluorescence data were collected each cycle after the 1 min step at 60°C. Amplification data were acquired through SDS Software (Applied Biosystems).

### Data Quality Control, Normalization, and Analysis

Obtained data fulfilled the criteria of PCR array reproducibility, RT efficiency, and genomic DNA contamination. If the average PPC Ct was 19 ± 3 and no two arrays had an average PPC Ct > 2 away from one another, then the samples passed the criteria for the PCR array reproducibility. If ΔCt (AVG RTC - AVG PPC) was ≤5, then the samples passed the criterion for RT efficiency. If Ct (MGDC) was ≥35, then the samples passed the criterion for genomic DNA contamination.

Three independent biological experiments were performed for each experimental group. The normalization was performed using the most stable genes/gene in the PCR array data set, identified by software at the Qiagen Data Analysis Center. The Ct values for these genes were geometrically averaged and used for the calculation of ΔΔCt values. The data are presented as fold change (2^-ΔΔCt^) which is the normalized gene expression (2^-ΔCt^) in the test sample divided by the normalized gene expression (2^-ΔCt^) in the control sample. Fold regulation represents fold change results in a biologically meaningful way. Fold change values greater than one indicate a positive- or an up-regulation, and the fold regulation is equal to the fold change. Fold change values less than one indicate a negative or down-regulation, and the fold regulation is the negative inverse of the fold-change. The *P*-values were calculated based on a Student’s *t*-test of the replicate 2^-Δ Ct^ values for each gene in the control group and treatment groups. Significance was assessed at ^∗^*P* ≤ 0.05 and ^∗∗^*P* ≤ 0.01.

### Immunophenotyping

Multi-color immunophenotyping was performed as previously described (Szulc-Dąbrowska et al., 2017). Cell surface maturation markers were stained with the following mAbs used in appropriate combinations: anti-H-2D[b]-PE (KH95, mouse IgG2b), anti-H-2D[d]-PE (34-2-12, mouse IgG2a), anti-I-A/I-E-BV711, anti-CD40-APC (3/23, rat IgG2a), anti-CD80-APC (16-10A1, Armenian hamster IgG2), anti-CD83-PE (Michel 19, rat IgG1) (all from BD Biosciences), and anti-CD86-PerCP-Cy5.5 (GL-1, rat IgG2a; eBioscience). Chemokine receptors were stained with anti-CD191(CCR1)-APC (643854, Rat IgG2b; R&D Systems), anti-CD195(CCR5)-PE (C34-3448, rat IgG2c; BD Biosciences), and anti-CD197(CCR7)-PerCP/Cy.5.5 (4B12, rat IgG2a; BioLegend) mAbs. Additionally, GM-BM were labeled for CD11c marker using anti-CD11c-BV421 mAbs. Appropriate isotype controls (purchased from BD Biosciences) were used to determine the percentages of cells expressing the respective markers. Moreover, to obtain proper gating strategies the Fluorescence Minus One samples were included as negative controls.

### Intracellular Staining

Non-, uvi-ECTV-, or ECTV-infected GM-BM were left unstimulated or were stimulated with LPS for 24 h in a 24-well plate at a density of 5 × 10^5^ cells/well. Brefeldin A (6 μg/ml; BD Biosciences) was added for the last 5 h of culture. Cells were then collected and stained with anti-CD11c-BV421. Intracellular staining of cytokines was performed using a Cytofix/Cytoperm kit (BD Biosciences) according to the manufacturer’s instructions. The following mAbs were used for cytokine detection: anti-tumor necrosis factor (TNF)-FITC (MP6-XT22, rat IgG1), anti-IL-12(p40/p70)-APC (C15.6, rat IgG1; both from BD Biosciences) and anti-CCL3 [macrophage inflammatory protein 1 alpha (MIP-1α)]-PE (DNT3CC; rat IgG2a; eBioscience, San Diego, CA, United States). In some experiments, cells were stained intracellularly for the presence of ECTV antigens using a Cytofix/Cytoperm kit and rabbit polyclonal Abs anti-ECTV-FITC, obtained as previously described (Szulc-Dabrowska et al., 2016). The staining procedure also included appropriate isotype controls obtained from BD Biosciences and eBioscience.

### Flow Cytometry Analysis

The population of live cells was gated based on size and granularity according to forward (FSC-A) and side (SSC-A) scatter profile. GM-BM enriched in cDC population were gated as FSC^high^ and CD11c^+^. Twenty thousand gated cell events were acquired from each specimen and analyzed on a BD FACSCanto II and BD LSRFortessa flow cytometers (Becton Dickinson). Data were analyzed with FACSDiva 7.0 software (Becton Dickinson).

### Enzyme-Linked Immunosorbent Assay

CD11c^+^-enriched GM-BM were plated into a 24-well plate at a density of 2 × 10^4^ cells/well. After treatment with medium (mock), uvi-ECTV, or live ECTV, cells were incubated with or without LPS for 4, 8, 12, 18, and 24 h. In some experiments, cells were additionally stimulated with 3 mM ATP for 6 h to induce IL-1β and IL-18 release (Mehta et al., 2000). The concentration of cytokines and chemokines in culture supernatants was quantified using available commercial sandwich enzyme-linked immunosorbent assay (ELISA), according to the instructions given by the manufacturers. TNF, IL-6, IL-10, IL-12p40, IL-12p70, and CCL2/monocyte *chemoattractant* protein 1 (MCP-1) were detected using BD OptEIA ELISA sets (BD Biosciences). CCL3/MIP-1α and CCL5/RANTES (regulated upon activation normal T cell expressed and secreted) were quantified using Quantikine ELISA Kits (R&D Systems). IL-18 and IL-15R/IL-15 complex were assessed using Platinum ELISA and ELISA Ready-SET-Go!, respectively (eBioscience). The absorbance was read at 450 nm using Epoch Microplate Spectrophotometer. Each plate contained its own standard curve for determination of cytokine/chemokine levels in the supernatants.

### Nitric Oxide detection

Nitric oxide (NO) formation can be investigated by measuring nitrite (NO_2_^-^), which is one of two primary, stable, and non-volatile breakdown products of NO. The amount of nitrite (μM/ml) in culture supernatants was assessed using the Griess Reagent System (Promega, Madison, WI, United States), according to the manufacturers’ protocol. Briefly, an equal volume of supernatant was mixed with the Sulfanilamide Solution and incubated for 10 min at room temperature in the dark. Then, *N*-1-naphthylethylenediamine dihydrochloride solution was added and incubated for additional 10 min. The absorbance was measured at 520 nm using Epoch Microplate Spectrophotometer. A standard curve generated with sodium nitrite was used to calculate the nitrite concentration in the supernatants.

### Statistical Analysis

Data are presented as mean ± standard deviation (SD) from at least three independent biological replicates. Normal distribution of variables was assessed using the Shapiro–Wilk *W*-test. If the data were normally distributed and the variances were homogeneous, then two-independent (unpaired) Student’s *t*-test was applied for group comparison. Some data were analyzed using two-dependent (paired) Student’s *t*-test. If the data were non-normally distributed, then the Wilcoxon signed-rank or Mann–Whitney *U*-tests were applied for comparison of paired and unpaired values, respectively (STATISTICA 6.0 software, StatSoft Inc., Tulsa, OK, United States). Statistical significance was assessed at ^∗^*P* ≤ 0.05 and ^∗∗^*P* ≤ 0.01.

## Results

### The Kinetics of ECTV Replication Cycle Are Similar in GM-BM Derived from C57BL/6 and BALB/c Mice

**FIGURE 1:**
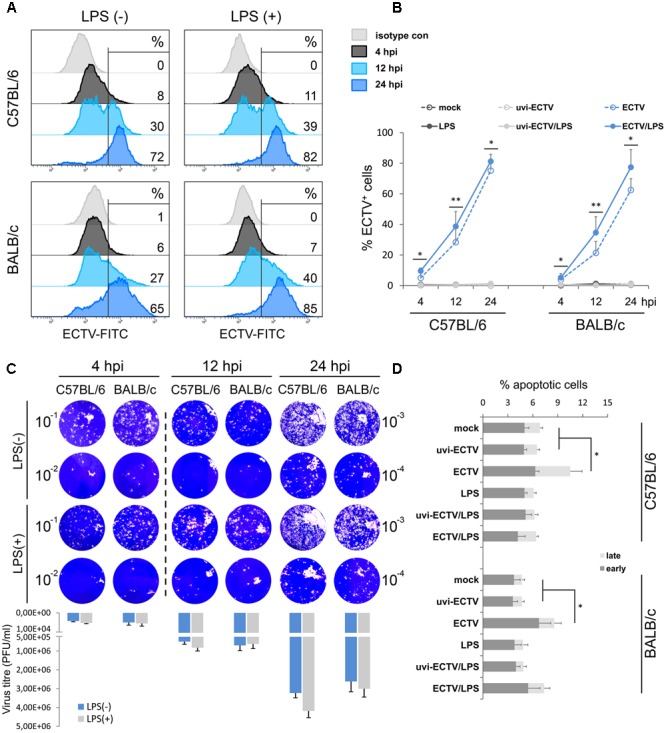
Similar kinetics of ECTV replication in GM-BM of C57BL/6 and BALB/c mice. **(A)** Representative flow cytometry histograms showing the percentage of ECTV^+^ cells at 4, 12, and 24 hpi of GM-BM. **(B)** The percentage (mean ± SD) of ECTV^+^ cells in GM-BM culture. **(C)** Plaque assay determination of ECTV titer in GM-BM at 4, 12, and 24 hpi. Plaques were visualized by staining of Vero cell monolayers with 0.3% crystal violet. The mean number of infectious virions (PFU/ml) was calculated from two experiments. **(D)** The percentage (mean ± SD) of early and late apoptotic cells in GM-BM during ECTV infection (Student’s *t*-test; ^∗^*P* < 0.05, ^∗∗^*P* < 0.01).

Our previous study showed that ECTV is able to productively infect murine GM-BM, including cDCs, with subsequent release of progeny virions (Szulc-Dąbrowska et al., 2017). In the present study, we compared the kinetics of ECTV replication cycle in GM-BM derived from resistant C57BL/6 and susceptible BALB/c mice. Cells were infected with ECTV at MOI = 1 and after 4, 12, and 24 hpi the percentage of ECTV^+^ cells was determined by intracellular staining and flow cytometry analysis (**Figure 1A**). The mean percentage of ECTV^+^ cells during the first 12 h of virus replication was comparable between both strains of mice (**Figure 1B**). At 24 hpi, the percentage of ECTV^+^ cells was slightly higher in C57BL/6 comparing to BALB/c mice (75 vs. 63%, respectively). LPS treatment of ECTV-infected GM-BM derived from both mouse strains significantly (*P* ≤ 0.05) increased the percentage of ECTV^+^ cells during the entire virus replication cycle. It suggests that TLR4-agonist stimulation positively regulates the tempo of virus reproduction in GM-BM infected at MOI of 1 (Szulc-Dąbrowska et al., 2017). Additionally, to confirm that ECTV infection of GM-BM is productive, we performed a plaque assay to determine quantify infectious viral particles (**Figure 1C**). At 4 hpi, the number of infectious virions was 4.2 × 10^3^ and 5 × 10^3^ PFU/ml in C57BL/6 and BALB/c GM-BM cultures, respectively. At 12 and 24 hpi, the virus titers in both cultures increased around 100- and 500-fold, respectively, and reached 5 × 10^5^ and 7 × 10^5^ PFU/ml at 12 hpi, and 3.2 × 10^6^ and 2.6 × 10^6^ PFU/ml at 24 hpi in C57BL/6 and BALB/c GM-BM cultures, respectively (**Figure 1C**). LPS treatment slightly increased the number of infectious virus particles, especially at 24 hpi and is agreement with flow cytometry results.

Our previous study also showed that 10-day culture of GM-BM and later infected with ECTV at MOI = 5 exhibited a high percentage (more than 30%) of early and late apoptotic cells during later stages of infection (Szulc-Dąbrowska et al., 2017). To minimize the apoptotic rate in GM-BM cultures, in the present study we used 8-day culture of GM-BM and later infected with ECTV at MOI = 1. Under such conditions, we observed only a small induction of apoptosis in GM-BM at 24 hpi in the absence of LPS (**Figure 1D**). Meanwhile, ECTV infection of GM-BM in the presence of LPS slightly (but not significantly) increased the percentage of apoptotic cells compared with LPS-treated mock- or uvi-ECTV-infected cells (**Figure 1D**). It is likely that LPS prevents the apoptotic effect induced by the virus infection, since LPS has been shown to inhibit caspase 3-dependent apoptosis in immune cells (Russe et al., 2014).

### Generalized Down-Regulation of Genes Involved in Innate and Adaptive Immune Functions Is Comparable in GM-BM-Derived from C57BL/6 and BALB/c Mice

**FIGURE 2:**
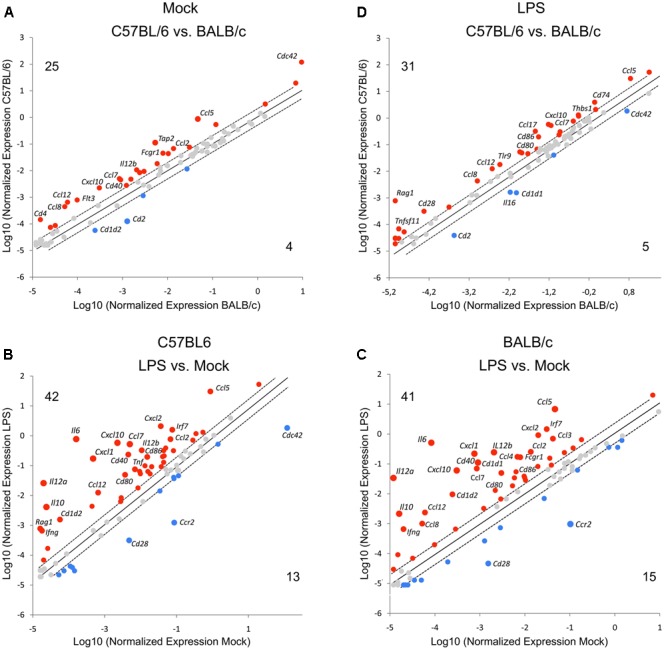
Differential expression of genes involved in innate and adaptive immune functions in GM-BM from C57BL/6 and BALB/c mice. The scatter plots of gene expression profiling in **(A)** mock-infected GM-BM from C57BL/6 vs. BALB/c mice, **(B)** LPS-treated vs. untreated mock-infected GM-BM from C57BL/6 mice, **(C)** LPS-treated vs. untreated mock-infected GM-BM from BALB/c mice, **(D)** mock-infected LPS-treated (for 24 h) GM-BM from C57BL/6 vs. BALB/c mice. The numbers represent genes that are up-regulated (red) and down-regulated (blue). The cut-offs for twofold induction and repression are indicated by dotted lines (all points having a fold-change less than 2 are shown in gray).

Before assessment of ECTV impact on gene expression profile, we first compared the gene expression between uninfected GM-BM derived from C57BL/6 and BALB/c mice to determine interstrain differences in genes involved in innate and adaptive immune functions (**Figure 2**). These genes, encoding proteins that regulate biological properties of DCs, were divided into seven categories: (1) antigen uptake, (2) antigen presentation, (3) chemotaxis, (4) chemokines and cytokines, (5) cytokine receptors, (6) other cell surface receptors, and (7) signal transduction.

Before calculating 2^-Δ ΔCt^ values, the data were normalized to the geometric mean of the four most stable housekeeping genes: *ActB, Gapdh, Gusb*, and *Hsp90ab1*. The scatter plots of the mRNA expression profiles of untreated and LPS-treated C57BL/6 vs. BALB/c GM-BM are presented in **Figures 2A,D**, respectively. The scatter plots compare the normalized expression of every gene on the array between two groups by plotting them against one another to quickly visualize large gene expression changes. As shown in **Figure 2A**, C57BL/6 compared to BALB/c GM-BM exhibited up-regulation of 25 genes, including *Ccl5, Ccl8, Ccl17, Cxcl10, Cd40, Fas, Fcer2a, Fcgr1, Il-12b, Irf7, Thbs1*, and *Tlr9*, and down-regulation of four genes, *Cd1d2, Cd2, Fcgrt*, and *Icam2*. LPS treatment of C57BL/6 and BALB/c GM-BM for 24 h resulted in up-regulation of 42 and 41 genes, and down-regulation of 13 and 15 genes, respectively (**Figures 2B,C**). Up-regulated genes were those known to influence maturation of DCs and included genes for cell surface receptors (e.g., *Cd1d1, Cd1d2, Cd40, Cd80, Cd86, H2-DMa, Tlr1, Tlr2*), chemokines and cytokines (e.g., *Ifn, Il10, Il12a, Il12b, Tnf, Ccl2, Ccl3, Ccl4, Ccl5, Ccl7, Ccl8, Ccl12, Ccl17, Ccl17, Cxcl1, Cxcl2, Cxcl10*) and signal transduction (e.g., *Irf7*). *Cd28, Cd36, Cd209a*, and *Clec4b2* were the commonly down-regulated genes after LPS exposure of GM-BM from both mouse strains. Interestingly, C57BL/6 GM-BM treated with LPS for 24 h showed up- and down-regulation of 31 and 5 genes, respectively, compared to BALB/c GM-BM treated with LPS (**Figure 2D**). Among up-regulated genes there were those primarily engaged in the antigen uptake (*Tap2*) and presentation (*Cd4, Cd40, Cd80, Cd86, H2-DMa, Thbs1*) and chemokine and cytokine production (*Ccl5, Ccl7, Ccl8, Ccl12, Cxcl2, Cxcl10, Cxcl12*). Moreover, TLR9 mRNA was significantly (*P* ≤ 0.01) up-regulated in C57BL/6 compared with BALB/c GM-BM after LPS-treatment. Collectively, it may suggest that C57BL/6 GM-BM are at a higher maturation state than BALB/c GM-BM after TLR4 agonist stimulation.

**FIGURE 3:**
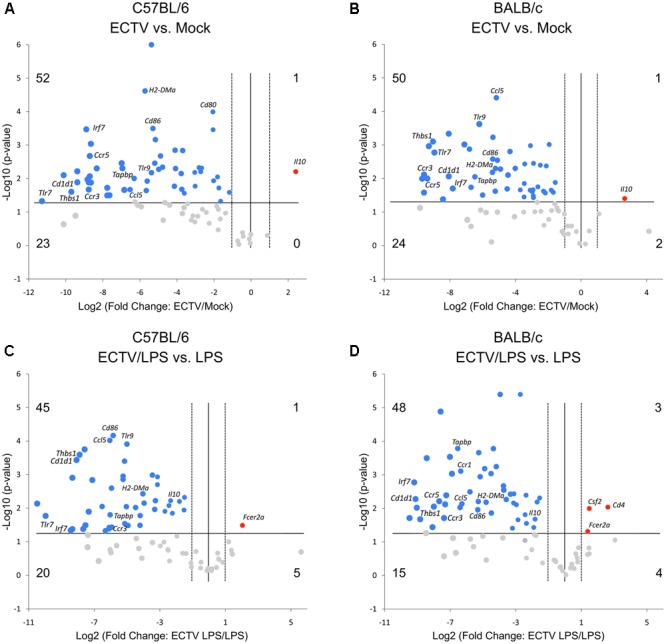
ECTV similarly affects multiple genes involved in innate and adaptive immune functions in GM-BM from C57BL/6 and BALB/c mice. The volcano plots show differentially expressed genes in ECTV- vs. mock-infected GM-BM from C57BL/6 **(A)** and BALB/c **(B)** mice, and ECTV + LPS- vs. LPS-treated GM-BM from C57BL/6 **(C)** and BALB/c **(D)** mice. The volcano plots display the threshold for statistical significance (log10 of the *P*-value; horizontal line) and fold change (log2; vertical dotted lines). The two vertical dotted lines represent fold changes of –2 and +2, the horizontal line represents significant *P*-value of 0.05. The number of genes that are down(blue)- and up(red)-regulated by at least twofold and have a *P*-value less than 0.05 are at the upper-left and upper-right, respectively. The number of genes that are down- and up-regulated (gray) by at least twofold, however, have a *P*-value more than 0.05 are at the lower-left and lower-right, respectively.

The gene expression comparison between uninfected and infected cells untreated or treated with LPS was made after data normalization to *Cdkn1a*, due to its uniform expression across treatment groups in the experiments. Differentially expressed genes are presented by volcano plots, which summarize fold-change in gene expression and statistical significance in four different experimental groups (**Figure 3**). ECTV infection of GM-BM derived from C57BL/6 and BALB/c mice resulted in a global down-regulation of 75 and 74 genes, respectively, among which 52 and 50 were significantly (*P* ≤ 0.05) repressed (**Figures 3A,B**). Profound inhibition in gene expression was observed in all gene categories in cells from both mouse strains. Only *Il10* gene was significantly (*P* ≤ 0.05) up-regulated in infected cells. Moreover, ECTV infection inhibited LPS-induced up-regulation of mRNA transcripts for many genes involved in activation and maturation of GM-BM. LPS-treated ECTV-infected cells derived from C57BL/6 and BALB/c mice displayed down-regulation of 65 and 63 genes, respectively, compared to LPS-treated uninfected cells (**Figures 3C,D**). Among these genes 45 and 48, respectively, were significantly (*P* ≤ 0.05) down-regulated. Taken together, our data clearly demonstrate that ECTV induces global gene repression in GM-BM of both strains and this repression is maintained during TLR4 agonist treatment.

### Expression of Genes Involved in Antigen Uptake and Presentation

**FIGURE 4:**
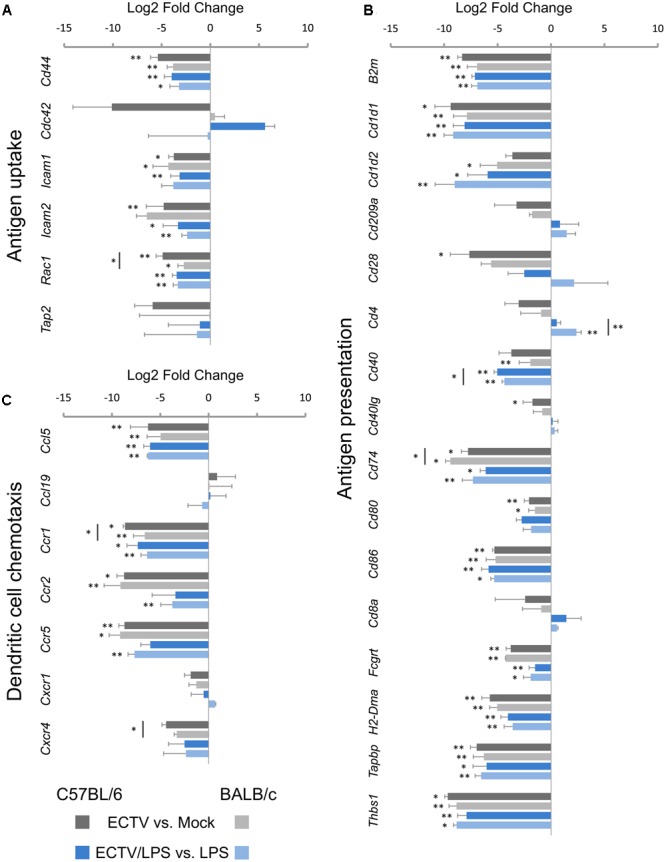
Mouse strain-independent effect of ECTV infection on the expression of genes involved in **(A)** antigen uptake, **(B)** antigen presentation, and **(C)** chemotaxis in GM-BM (unpaired Student’s *t*-test; ^∗^*P* < 0.05, ^∗∗^*P* < 0.01).

In the antigen uptake category, *Cd44, Icam1, Icam2*, and *Rac1* were mostly significantly (*P* ≤ 0.05) down-regulated in untreated or LPS-treated ECTV-infected C57BL/6 and BALB/c GM-BM in comparison with their uninfected counterparts (**Figure 4A**). The mean expression level of these genes was more than 10-fold repressed. The level of *Rac1* repression was significantly (*P* ≤ 0.05) higher in infected C57BL/6 than BALB/c cells. Meanwhile, *Cdc42* was not significantly up-regulated in LPS-treated ECTV-infected GM-BM from C57BL/6 mice compared to LPS-treated uninfected cells (**Figure 4A**).

Of the 16 genes analyzed in the antigen presentation category, ECTV infection resulted in significantly (*P* ≤ 0.05) down-regulation of 11 mRNA transcripts in C57BL/6 and BALB/c GM-BM (**Figure 4B**). *B2m, Cd1d1, Cd74, Cd80, Cd86, Fcgrt, H2-DMa, Tapbp*, and *Thbs1* were significantly (*P* ≤ 0.05) repressed in cells from both mouse strains, whereas *Cd28, Cd40Ig*, and *Cd1d2, Cd40* were significantly down-regulated in C57BL/6 and BALB/c GM-BM, respectively. The level of *Cd74* repression was significantly (*P* ≤ 0.05) higher in infected BALB/c than C57BL/6 cells. LPS treatment of mock-infected C57BL/6 and BALB/c GM-BM resulted in up-regulation of the following genes: *Cd1d1, Cd1d2, Cd209a, Cd40, Cd80, Cd86, Tapbp*, and *Thbs1*. Meanwhile, treatment of ECTV-infected cells with LPS did not up-regulate genes, as observed in mock-infected LPS-treated GM-BM from the two mouse strains. After stimulation with LPS, infected C57BL/6 and BALB/c GM-BM still exhibited a profound down-regulation of 10 following genes: *B2m, Cd1d1, Cd1d2, CD40, Cd74, Cd86, Fcgrt, H2-DMa, Tapbp*, and *Thbs1*. In all cases the most repressed genes were *Cd1d1* and *Thbs1*, and their mean expression levels were ≥300-fold decreased. The repression level of mRNA transcript for CD40 was significantly (*P* ≤ 0.05) higher in LPS-stimulated ECTV-infected C57BL/6 than BALB/c cells. The mRNA expression of CD80 was not significantly (*P* > 0.05) down-regulated (**Figure 4B**). Only *Cd4* was differentially regulated in LPS-treated ECTV-infected GM-BM from the two strains of mice. *Cd4* was significantly (*P* = 0.009) up-regulated in BALB/c cells, whereas in C57BL/6 cells its expression remained unchanged.

### Expression of Genes Involved in DC Chemotaxis

The four important genes in this group were significantly (*P* ≤ 0.05) down-regulated in ECTV-infected GM-BM from both mouse strains (**Figure 4C**). These genes were *Ccl5, Ccr1, Ccr2*, and *Ccr5*. *Ccr1* was significantly (*P* ≤ 0.05) more repressed in infected C57BL/6 than BALB/c cells. The same set of genes were also down-regulated in LPS-treated ECTV-infected GM-BM compared to LPS-treated uninfected cells. However, in cells from C57BL/6 mice, the repression of *Ccr2* and *Ccr5* was not statistically significant (*P* > 0.05). Although the down-regulation of *Cxcr4* in GM-BM from both mouse strains was not significant, statistical analysis revealed that this gene was considerably repressed in C57BL/6 mice.

### Expression of Genes Involved in Chemokine and Cytokine Production

**FIGURE 5:**
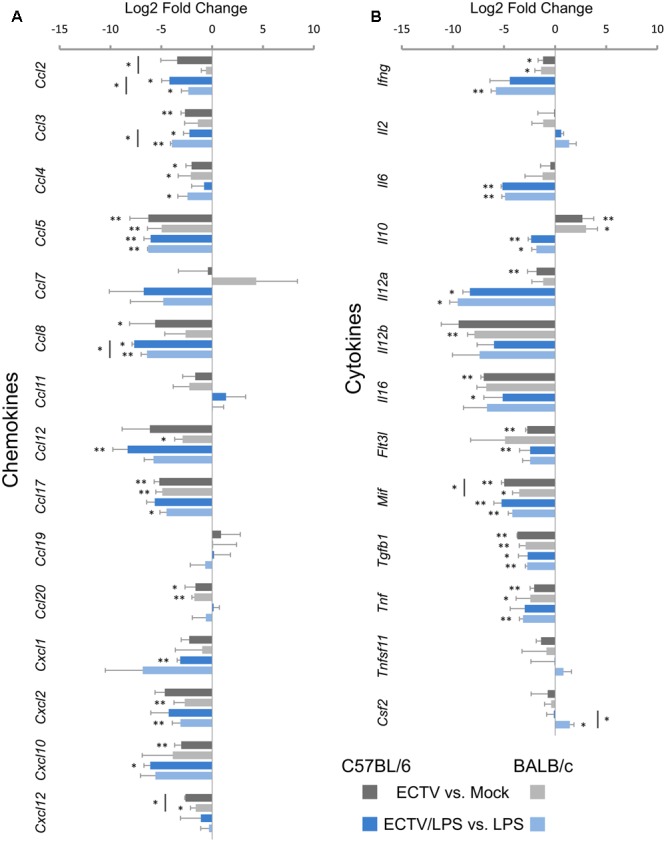
Mouse strain-independent effect of ECTV infection on the expression of genes for chemokines **(A)** and cytokines **(B)** in GM-BM (unpaired Student’s *t*-test; ^∗^*P* < 0.05, ^∗∗^*P* < 0.01).

The next category of analyzed genes concerned those engaged in the synthesis of chemokines (**Figure 5A**) and cytokines (**Figure 5B**). Among the 15 chemokine genes assayed, seven genes in both C57BL/6 and BALB/c GM-BM were significantly (*P* ≤ 0.05) down-regulated (**Figure 5A**) following ECTV infection. *Ccl4, Ccl5, Ccl17*, and *Ccl20* were repressed in cells from both mouse strains, whereas *Ccl2, Ccl3, Ccl8* and *Ccl12, Cxcl2, Cxcl12* were decreased in C57BL/6 and BALB/c GM-BM, respectively (**Figure 5A**). LPS treatment of mock-infected cells up-regulated 12 genes (*Ccl2, Ccl3, Ccl4, Ccl5, Ccl7, Ccl8, Ccl12, Ccl17, Ccl20, Cxcl1, Cxcl2*, and *Cxcl10*) in GM-BM from both mouse strains (**Figures 2C,D**). Meanwhile, LPS-treated ECTV-infected cells showed significant (*P* ≤ 0.05) down-regulation of seven mRNA transcripts compared to LPS-treated uninfected cells (**Figure 5A**). GM-BM from both mouse strains exhibited repression of *Ccl2, Ccl3, Ccl5*, and *Ccl8*, whereas *Ccl12, Cxcl1, Cxcl10* and *Ccl4, Ccl17, Cxcl2* were significantly (*P* ≤ 0.05) decreased in cells from C57BL/6 and BALB/c mice, respectively. ECTV-infected C57BL/6 GM-BM displayed significant (*P* ≤ 0.05) down-regulation of *Ccl2, Cxcl12* and *Ccl2, Ccl3, Ccl8* than BALB/c cells, cultured without or with LPS, respectively.

Among the 13 genes analyzed in the cytokine group, *Ifng, Mif, Tgfb1*, and *Tnf* were significantly (*P* ≤ 0.05) repressed in GM-BM of both mouse strains (**Figure 5B**). The level of *Mif* expression was significantly (*P* ≤ 0.05) decreased in C57BL/6 cells. *Il12a, Il16*, and *Flt3l* were also down-regulated, however, significant (*P* ≤ 0.05) repression of these genes was observed only in C57BL/6 cells. On the contrary, the expression of *Il12b* was significantly (*P* ≤ 0.01) decreased in BALB/c GM-BM. Interestingly, ECTV infection significantly (*P* ≤ 0.05) up-regulated the expression of *Il10* in cells from both mouse strains (**Figure 5B**).

Uninfected GM-BM from C57BL/6 and BALB/c mice displayed up-regulation of *Ifng, Il6, Il10, Il12a, Il12b*, and *Tnf* after LPS treatment for 24 h (**Figures 2C,D**). Meantime, LPS-stimulated ECTV-infected cells exhibited significant (*P* ≤ 0.05) repression of *Il6, Il10, Il12a, Mif*, and *Tgfb1*, compared to uninfected cells incubated with TLR4 agonist (**Figure 5B**). Moreover, LPS-treated ECTV-infected GM-BM from C57BL/6 and BALB/c mice showed down-regulation of *Il16* and *Flt3l* or *Ifng* and *Tnf*, respectively. *Csf2* was significantly up-regulated only in BALB/c GM-BM after ECTV infection and LPS treatment.

### Expression of Genes for Cytokine and Other Cell Surface Receptors

**FIGURE 6:**
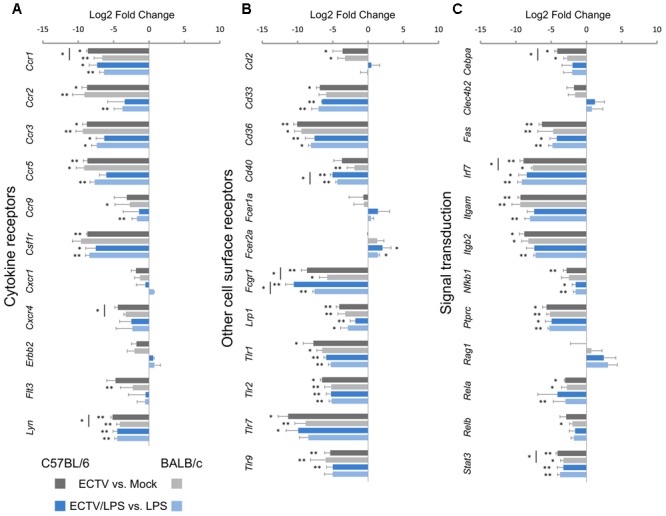
ECTV similarly affects genes for **(A)** cytokine receptors and **(B)** other cell surface receptors, and **(C)** genes involved in signal transduction in GM-BM from C57BL/6 and BALB/c mice (unpaired Student’s *t*-test; ^∗^*P* < 0.05, ^∗∗^*P* < 0.01).

In the cytokine receptors category, *Ccr1, Ccr2, Ccr3, Ccr5, Csf1r*, and *Lyn* were significantly (*P* ≤ 0.05) down-regulated in untreated or LPS-treated ECTV-infected GM-BM from both mouse strains, compared to their uninfected counterparts (**Figure 6A**). *Ccr1, Cxcr4*, and *Lyn* were significantly (*P* ≤ 0.05) more repressed in C57BL/6 than BALB/c GM-BM. Moreover, *Ccr9* and *Flt3* were significantly decreased only in infected BALB/c cells.

The majority of other analyzed genes in the cell surface receptors category were down-regulated in ECTV-infected GM-BM from both mouse strains (**Figure 6B**). *Cd36, Fcgr1, Lrp1, Tlr1*, and *Tlr2* were significantly (*P* ≤ 0.05) repressed in cells from both mouse strains and such inhibitory effect was independent of LPS stimulation. *Cd2, Tlr7*, and *TLr9* were also down-regulated in both strains, however, after LPS stimulation *Tlr7* and *TLr9* were significantly (*P* ≤ 0.05) repressed only in C57BL/6 GM-BM. On the contrary, *Fcer2a* was significantly (*P* ≤ 0.05) up-regulated in ECTV-infected C57BL/6 and BALB/c GM-BM treated with LPS (**Figure 6B**). LPS stimulation of mock-infected cells resulted in up-regulation of *Cd40, Fcgr1, Tlr1*, and *Tlr2* (**Figures 2C,D**).

### Expression of Genes Involved in Signal Transduction

The last analyzed group concerned 12 genes involved in signal transduction (**Figure 6C**). The following 8 genes were mostly significantly (*P* > 0.05) down-regulated in ECTV-infected GM-BM derived from both strains of mice and independent of LPS treatment: *Fas, Irf7, Itgam, Itgb2, Nfkb1, Ptprc, Rela*, and *Stat3*. Moreover, *Cebpa* and *Relb* were significantly (*P* > 0.05) down-regulated in GM-BM from both and only BALB/c mouse, respectively. *Cebpa, Irf7* and *Stat3* were significantly (*P* > 0.05) more repressed in infected C57BL/6 than BALB/c GM-BM (**Figure 6C**).

### Mouse Strain-Independent Effect of ECTV-Infection on Cytokine and NO Production by GM-BM

cDCs play a key role in driving T cell responses by eliciting polarizing signals, the most important of which are cytokines that selectively promote the generation of Th1 or Th2 cells. We next confronted the GM-BM obtained from resistant C57BL/6 and susceptible BALB/c mice regarding their Th-polarizing cytokine profile under ECTV infection *in vitro*. The analyzed cytokines and chemokines are engaged in T cell activation and involved in regulation of Th1 or Th2 polarization during the adaptive immune responses.

**FIGURE 7:**
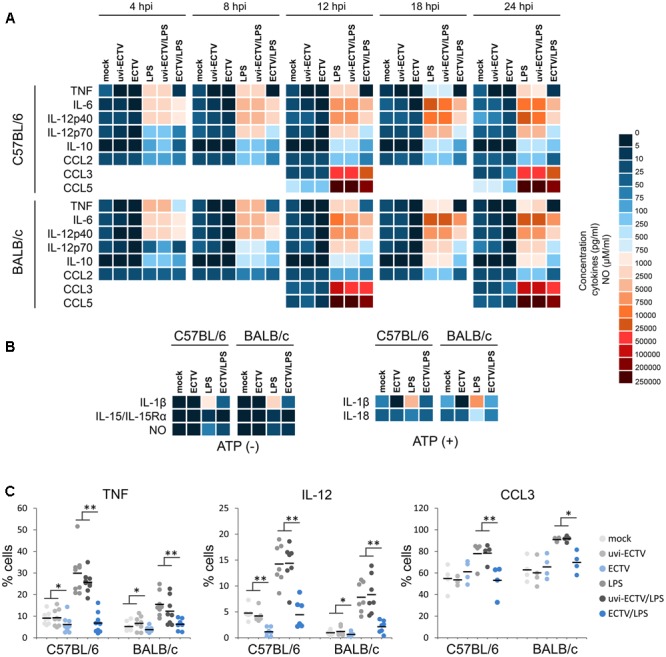
The effect of ECTV infection on cytokine and chemokine synthesis and NO production by GM-BM is mouse strain-independent. Heat map represents mean concentration (pg/ml) values of cytokines and chemokines in culture supernatants of mock-, uvi-ECTV-, and ECTV-infected GM-BM, untreated or treated with LPS **(A)**, or additionally with ATP **(B)**. Mean NO concentration is presented as heat map ranging from 0 to 100 μM/ml **(B)**. **(C)** The percentage of cytokine-producing GM-BM at 24 hpi with ECTV. Each point represents an individual data, with the bar indicating the mean values (paired Student’s *t*-test; ^∗^*P* < 0.05, ^∗∗^*P* < 0.01).

TNF-α contributes to DC activation and maturation and is required for subsequent induction of optimal T cell responses (Zhang et al., 2003). Uninfected GM-BM from C57BL/6 and BALB/c mice produced TNF-α at similar low levels, however, after LPS stimulation, BALB/c cells secreted higher amounts of TNF-α than C57BL/6 cells (**Figure 7A**). During LPS treatment, the concentration of TNF-α in culture supernatants from C57BL/6 and BALB/c GM-BM started to decrease at 24 h post-stimulation and was two- and fourfold lower, respectively, than at 4 h post-stimulation. Meantime, ECTV infection of GM-BM from both strains of mice profoundly inhibited TNF-α secretion, even after LPS treatment.

IL-6 produced by DCs in response to TLR recognition of microbial products has been demonstrated to regulate T cell activation by overcoming the suppressive effect of Tregs (Pasare and Medzhitov, 2003). Similar to TNF-α, mock- or uvi-ECTV-infected GM-BM from both mouse strains secreted comparable low levels of IL-6. After LPS stimulation, GM-BM of both strains of mice produced high amounts of IL-6 and from 8 h post-stimulation, DCs from BALB/c mice produced higher levels of IL-6 than those from C57BL/6 mice (**Figure 7A**). ECTV-infected GM-BM untreated or treated with LPS were able to secrete IL-6, however, the amounts were significantly (*P* > 0.01) lower compared to those secreted by mock- or uvi-ECTV-infected LPS-untreated or -treated GM-BM.

Next, we analyzed cytokines and chemokines engaged in Th1 polarization, such as: IL-12p40, IL-12p70, IL-15/IL-15Rα, IL-18, CCL3/MIP-1α, and CCL5/*RANTES* (**Figures 7A,B**). C57BL/6 GM-BM produced larger amounts of IL-12p40 and IL-12p70 than BALB/c GM-BM, especially after LPS treatment (**Figure 7A**). ECTV-infection significantly (*P* > 0.01) decreased the production of both cytokines by LPS-untreated or -treated GM-BM of both mouse strains. Meantime, CCL3 was secreted at higher levels by uninfected BALB/c than C57BL/6 cells, especially in response to the ligand for TLR4 (**Figure 7A**). At 12 hpi with ECTV, GM-BM from both mouse strains produced less CCL3 than mock- or uvi-ECTV infected cells, whereas at 24 hpi those cells showed significantly (*P* > 0.01) increased level of CCL3 compared to mock- or uvi-ECTV-treated cells. However, GM-BM infected with live ECTV exhibited the suppression of TLR4 agonist-induced secretion of CCL3. CCL5 production was higher by C57BL/6 GM-BM, however, LPS stimulation induced robust secretion of comparable large amounts of CCL5 by cells from both mouse strains (**Figure 7A**). The secretion of CCL5 was >2-fold reduced in ECTV-infected C57BL/6 and BALB/c GM-BM, either in the absence or presence of LPS.

IL-15/IL-15Rα complex was produced at similar levels by C57BL/6 and BALB/c GM-BM, even after LPS-stimulation for 24 h (**Figure 7B**). ECTV infection significantly (*P* > 0.01) reduced the level of this complex in GM-BM from both mouse strains at 24 hpi. IL-18 was also secreted at comparable amounts by uninfected cells from both strains of mice, however, after stimulation with LPS for 24 h and ATP for additional 6 h, BALB/c GM-BM produced fourfold higher levels of IL-18 than C57BL/6 GM-BM (**Figure 7B**). ECTV-infection significantly (*P* > 0.01) inhibited TLR4 agonist + ATP-induced IL-18 production.

Because NO selectively enhances Th1 cell proliferation (Niedbala et al., 1999), we additionally checked the influence of ECTV infection on NO production by GM-BM from C57BL/6 and BALB/c mice (**Figure 7B**). Uninfected GM-BM from both mouse strains produced low levels of NO. After LPS treatment for 24 h, cells produced elevated levels of NO. In addition, after TLR4 agonist stimulation C57BL/6 GM-BM produced larger amounts of NO than BALB/c GM-BM. However, ECTV-infected cells from both mouse strains exhibited a significant (*P* > 0.01) decrease in NO production in response to LPS stimulation.

Additionally to Th1-polarizing cytokines, we evaluated the effect of ECTV infection on GM-BM capacity to produce cytokines that positively regulate Th2 polarization: IL-10, CCL2/MCP-1 and IL-1β, which has also Th1-polarizing properties (**Figures 7A,B**). IL-10 was undetectable in mock-, uvi-ECTV-, or ECTV-infected cultures of GM-BM from both mouse strains, indicating that ECTV is not able to stimulate IL-10 secretion by these cells (**Figure 7A**). After LPS treatment, uninfected BALB/c GM-BM produced twofold higher amounts of IL-10 than C57BL/6 cells. ECTV infection significantly (*P* > 0.01) decreased the production of IL-10 by LPS-treated GM-BM from both mouse strains. CCL2 was secreted at similar levels by C57BL/6 and BALB/c GM-BM, however, after LPS treatment cells from C57BL/6 mice produced slightly more CCL2 compared to BALB/c GM-BM (**Figure 7A**). ECTV infection significantly (*P* > 0.01) decreased the level of CCL2 secreted by GM-BM from both strains of mouse in response to LPS. Meanwhile, BALB/c GM-BM produced more IL-1β compared to C57BL/6 cells, especially after LPS treatment (**Figure 7B**). Further stimulation of cells with ATP additionally increased the level of secreted IL-1β. Similar to other cytokines, ECTV infection significantly (*P* > 0.01) reduced the level of IL-1β in supernatants of GM-BM from both mouse strains cultured under different conditions.

Intracellular staining of selected cytokines revealed that ECTV-infection also reduced the percentage of cells producing TNF-α and IL-12p40/p70 (**Figure 7C**). After LPS treatment the percentage of GM-BM from both mouse strains producing TNF-α, IL-12p40/p70, and CCL3 was significantly (*P* > 0.05) decreased. Uninfected GM-BM from C57BL/6 and BALB/c mice produced more TNF-α and IL-12p40/p70 or CCL3, respectively, in response to LPS treatment. Collectively, our data indicate that C57BL/6 and BALB/c GM-BM are able to produce higher amounts of Th1- and Th2-polarizing cytokines, respectively, especially in response to TLR4 agonist stimulation. ECTV infection causes a profound inhibition of the production of both Th1 and Th2-polarizing cytokines by GM-BM in a strain-independent manner, indicating that there is no differences in the reactivity of cDCs from resistant C57BL/6 and susceptible BALB/c mice to the virus.

### The *in Vitro* Effect of ECTV-Infection on the Expression of MHC and Costimulatory Molecules on GM-BM Is Mouse Strain-Independent

**FIGURE 8:**
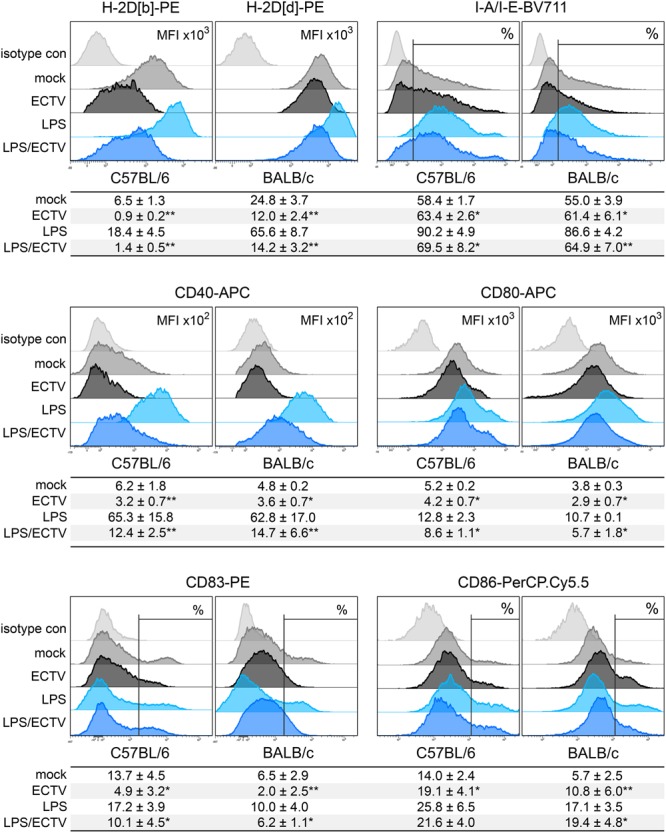
ECTV infection inhibits maturation of GM-BM in a mouse strain-independent manner. Representative flow cytometry histograms showing the effect of ECTV infection and/or LPS treatment on maturation marker expression in GM-BM from C57BL/6 and BALB/c mice at 24 hpi. Below each histogram set is a table with mean values (±SD) for a given marker from at least three independent experiments. The expression is presented as mean fluorescent intensity (MFI) or the percentage of positive cells for a given marker (paired Student’s *t*-test; ^∗^*P* < 0.05, ^∗∗^*P* < 0.01).

We compared the influence of ECTV infection on the expression of MHC class I and II molecules, as well as costimulatory molecules, including CD40, CD80, and CD86, and CD83, on GM-BM generated from bone marrow progenitor cells of different mouse strains (**Figure 8**). GM-BM derived from C57BL/6 mice expressed lower levels of H-2Db molecules than BALB/c cells H-2Dd molecules. However, C57BL/6 cells expressed higher level of I-A/I-E, CD40 and CD80 molecules than those from BALB/c mice. Moreover, a higher percentage of GM-BM expressing CD83 and CD86 molecules was found in GM-BM derived from C57BL/6 than BALB/c mice (**Figure 8**). This suggests that GM-BM derived from C57BL/6 mice mature more efficiently than those from BALB/c mice. LPS treatment increased the expression of all tested molecules on GM-BM from both mouse strains in a maturation-dependent manner (**Figure 8**).

ECTV infection modulated the expression of all tested maturation molecules on GM-BM from both mouse strains. Infected cells from C57BL/6 and BALB/c mice showed significantly (*P* > 0.01) reduced mean fluorescent intensity (MFI) of H-2Db and H-2Dd molecules, respectively, even after LPS treatment (**Figure 8**). The level of MFI for H-2D molecules was significantly more decreased in infected C57BL/6 compared to infected BALB/c cells, untreated (sevenfold vs. twofold; *P* = 0.0015) or treated (15-fold vs. 5-fold; *P* = 0.0024) with LPS. Meanwhile, in ECTV-infected cells we observed a significant (*P* ≤ 0.05) increase in the percentage of I-A/I-E-positive cells, however, after LPS stimulation the percentage of such cells was reduced compared to mock-infected LPS-treated cells. Additionally, ECTV infection significantly (*P* ≤ 0.05) reduced the expression of CD40 and CD80 costimulatory molecules on GM-BM from both mouse strains, even in the presence of LPS. Moreover, upon infection the percentage of CD83^+^ cells significantly (*P* ≤ 0.05) decreased in C57BL/6 and BALB/c GM-BM cultures. On the contrary, the percentage of CD86^+^ cells was significantly (*P* ≤ 0.05) increased in ECTV-exposed compared to mock-exposed cells from both mouse strains and in BALB/c cells treated with ECTV + LPS compared to cells treated only with LPS (**Figure 8**).

### Chemokine Receptor Expression in Down-Regulated in ECTV-Infected GM-BM from C57BL/6 and BALB/c GM-BM

**FIGURE 9:**
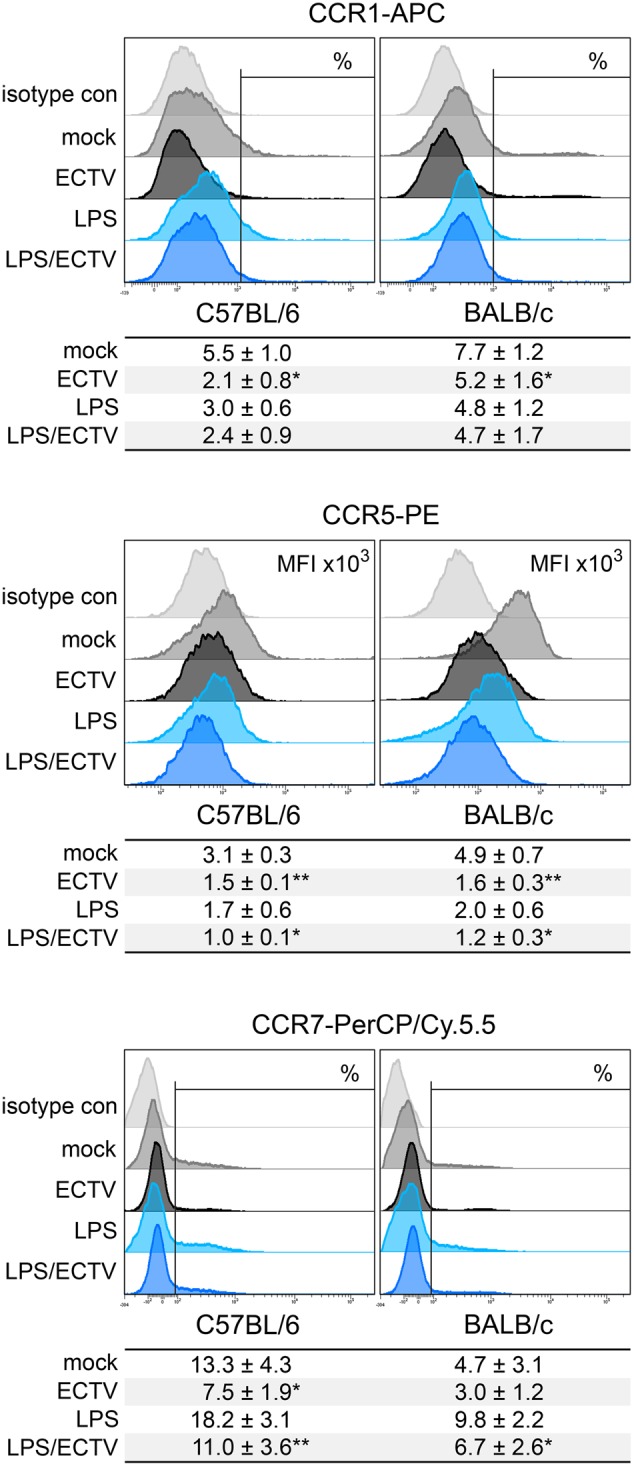
ECTV infection similarly affects the expression of chemokine receptors on GM-BM from C57BL/6 and BALB/c mice. Representative flow cytometry histograms of chemokine receptor expression on GM-BM following the infection with ECTV and/or treatment with LPS at 24 hpi. Below each histogram set is a table with mean values (±SD) for a given marker from at least three independent experiments. The expression is presented as mean fluorescent intensity (MFI) or the percentage of positive cells for a given marker. Statistical comparisons were between mock- and ECTV-exposed DCs and between LPS- and ECTV + LPS-exposed DCs (paired Student’s *t*-test; ^∗^*P* < 0.05, ^∗∗^*P* < 0.01).

Our last question concerned the effect of ECTV infection on the expression of cell surface chemokine receptors that are differentially regulated upon maturation. CCR1 and CCR5 are reported to be reduced, and CCR7 is reported to be up-regulated on the surface of mature DCs (Le Nouën et al., 2011). Our results show that GM-BM from BALB/c mice expressed higher levels of chemokine receptors characteristic for immature DCs, compared to C57BL/6 cells (**Figure 9**). BALB/c cultures contained a higher percentage of CCR1^+^ cells and expression of CCR5 on their surface was increased. On the contrary, C57BL/6 cultures had a higher percentage of CCR7^+^ GM-BM. After infection with ECTV, cells from both mouse strains exhibited reduced percentage of CCR1^+^ and CCR7^+^ cells and decreased expression of CCR5, compared to mock-infected cells. After LPS treatment, the expression of chemokine receptors changed in a maturation dependent manner, i.e., the percentage of CCR1^+^ and CCR7^+^ cells decreased and increased, respectively, and MFI for CCR5 was low. Meanwhile, stimulation of ECTV-infected GM-BM with LPS resulted in the reduction of the percentage of CCR7^+^ cells and MFI for CCR5 expression, compared to LPS-treated cells. Taken together, our results indicate that ECTV impairs expression of chemokine receptors on GM-BM and, therefore, it is not excluded that their potential to respond to chemokines regulating their migration is limited.

## Discussion

The inter-strain differences in the reactivity of DCs to the antigen exposure/infection contributes to various types of adaptive immune responses and may partially determine the outcome of a disease (Liu et al., 2000, 2002). Therefore, in the present study, we investigated the differences in the response of C57BL/6 and BALB/c GM-BM to ECTV infection by analysis of their maturation degree, chemokine receptor expression and production of different types of cytokines/chemokines engaged in T cell activation, polarization, and function. In addition, we analyzed the expression of key genes involved in maturation and activation of DCs. We found that *in vitro* ECTV modulation of GM-BM innate and adaptive immune properties occurred independently of the mouse strain susceptibility to infection. In cells from both strains of mice, ECTV infection contributed to the profound suppression of *polarizing signals to prime* Th1 immune response. Furthermore, contrary to expectations, a more pronounced viral inhibitory effect was observed in resistant C57BL/6 GM-BM, despite their higher potential to stimulate Th1 response under physiological conditions. These results indicate strong adaptation capacity of ECTV to the natural host DCs and its direct inhibitory effect on DC properties is irrespective of whether they are derived from resistant or susceptible mouse strain.

Several *in vivo* studies have reported that Th1 and Th2 immune responses prevail in C57BL/6 and BALB/c mice, respectively, under physiological conditions (Trunova et al., 2011), stress (Palumbo et al., 2010), or infection (Belkaid et al., 2002; Sacks and Noben-Trauth, 2002; Chaudhri et al., 2004; Watanabe et al., 2004). The immune response of different types of cells from these mouse strains, including DCs and macrophages, may influence the development of Th1 and Th2 adaptive immunity (Liu et al., 2002; Watanabe et al., 2004). It has been shown that DCs from C57BL/6 mice produce higher levels of IL-12 and IL-15 than DCs from BALB/c mice during early stages of *Listeria monocytogenes* infections (Liu et al., 2000). Moreover, DCs isolated from spleens of naïve C57BL/6 mice preferentially expressed a higher level of TLR9 mRNA, whereas those from BALB/c mice expressed a higher level of mRNAs for TLR2, -4, -5, and -6. In response to microbial ligands for TLR2 (lipoprotein), TLR4 (LPS), TLR2/6 (zymosan), and TLR9 (CpG), DCs from C57BL/6 and BALB/c mice produced larger amounts of IL-12p40 and CCL2, respectively. Additionally, DCs from C57BL/6 mice exhibited a more mature phenotype than BALB/c DCs due to higher expression of maturation markers, such as CD40 and CD86, and signal transducer and activator of transcription 4 (Stat4)—a key intermediate in IL-12 signaling pathway and Th1 cell development (Liu et al., 2002).

Our *in vitro* studies revealed that GM-BM derived from C57BL/6 mice displayed higher expression of *Ccl5, Cd40, Il-12b, Irf7, Thbs1*, and *Tlr9* than BALB/c cells. Moreover, upon LPS stimulation, *Cd80, Cd86, Ccl5*, and *Tlr9* were more up-regulated in C57BL/6 than BALB/c GM-BM. Additionally, C57BL/6 cells produced higher levels of IL-12p40 and IL-12p70, whereas BALB/c cells secreted more TNF-α, IL-6, and IL-10, especially after LPS treatment. Higher expression of maturation markers, such as MHC II, CD40, and CD80, and large percentage of cells expressing CD83, CD86, and CCR7 was observed in C57BL/6 cultures. Overall, our data indicate that GM-BM from resistant C57BL/6 mice are more matured than cells from susceptible BALB/c cells and possess a higher potential to stimulate Th1 response. Inter-strain differences in maturation state were also observed between GM-BM from BALB/c, C57BL/6, and atopic prone NC/Nga mice (Koike et al., 2008). The latter had greater expression of MHC class II, CD80, CD86, and CD11c compared to cells from BALB/c and C57BL/6 mice, as well as preferentially stimulated Th2 cytokine immune response. Therefore, it is suggested that the genetic background may enhance the differentiation and function of DCs and may be partially related to the development or aggravation of allergic/atopic diseases (Koike et al., 2008). On the contrary, resting BMDCs generated with GM-CSF and IL-4 from bone marrow progenitor cells of C57BL/6 and BALB/c mouse strains displayed no differences in the expression of CD40, CD80, CD86, MHC II, and TLR2 (Jiang et al., 2010).

It has been proposed that differences in innate and adaptive immune functions of DCs underline additional mechanisms responsible for resistance or susceptibility of C57BL/6 or BALB/c mice to *L. monocytogenes* (Liu et al., 2000, 2002). Moreover, DC capacity to differentiate naive T cells into functional Th1 or Th2 effector cells during *Leishmania major* infection is cell-intrinsic and Th2 polarization is not restricted to H2-d mice, since DCs from BALB/c and B10.D2 DCs (both H2d) exhibited differences in maturation and ability to induce Th differentiation (Filippi et al., 2003). In our *in vitro* study, the influence of ECTV infection on C57BL/6 and BALB/c GM-BM functions was comparable. Cells from both strains of mice exhibited a profound immunosuppression due to the productive virus replication (Szulc-Dąbrowska et al., 2017). Interestingly, GM-BM from resistant C57BL/6 mice had a higher percentage of ECTV^+^ cells compared to BALB/c cells at 24 hpi, suggesting that the viral spread is more efficient in these cells at later stages of infection. Moreover, upon ECTV infection 11 genes (*Rac1, Ccl2, Cxcl12, Mif, Ccr1, Cxcr4, Lyn, Fcgr1, Cebpa, Irf7*, and *Stat3*) were significantly more repressed in C57BL/6 cells compared to BALB/c cells, whereas the latter only had two genes (*Ccl3* and *Cd74*) more down-regulated compared to C57BL/6 cells. Additionally, infected GM-BM from C57BL/6 mice displayed a more expressive reduction in MHC I expression than BALB/c GM-BM. It is not excluded that slightly increased virus inhibitory effect on C57BL/6 GM-BM function is associated with a higher rate of ECTV dissemination within these cells. The ability of ECTV to replicate productively in GM-BM is a manifestation of its high adaptation capacity to the natural host immune cells, since other orthopoxviruses, such as CPXV and VACV abortively infect human DCs (Engelmayer et al., 1999; Jenne et al., 2000; Hansen et al., 2011).

Our studies revealed that ECTV infection in GM-BM derived from C57BL/6 and BALB/c mice down-regulated many genes involved in antigen uptake and processing, chemokine and cytokine synthesis, receptor expression, and signal transduction. The inhibitory effect on gene expression was also observed after TLR4 agonist treatment. In general, the analyzed genes were regulated in the same way in cells from both strains of mice and we did not observe any strain-specific response in GM-BM upon *in vitro* ECTV-infection. On the contrary, peritoneal macrophages from C57BL/6 and BALB/c mice infected with ECTV exhibited differential regulation of 14 innate antiviral genes, which were up-regulated in C57BL/6 and down-regulated in BALB/c cells, suggesting that these variations in gene expression may partially contribute to resistance or susceptibility to severe mousepox (Dolega et al., 2017). Meantime, several inter-strain variations in GM-BM functionality have been observed during paramyxovirus simian virus 5 (SV5) infection (Pejawar et al., 2005). Firstly, GM-BM from C57BL/6 mice were much more permissive to SV5 infection than cells derived from BALB/c mice. Secondly, despite the production of a similar panel of cytokines, cells differed in the maturation state: C57BL/6 cells up-regulated the expression of CD40, CD80, and CD86, whereas BALB/c cells displayed increase only in CD40 and CD86 expression. Thirdly, SV5-matured C57BL/6 GM-BM were more potent to activate naïve CD8^+^ T cells than SV5-matured BALB/c cells (Pejawar et al., 2005). Similar inter-strain differences were observed in BMDCs during infection with *Chlamydia muridarum* (Jiang et al., 2010). After *in vitro* infection, BMDCs from C57BL/6 mice underwent higher functional maturation than cells from BALB/c mice. This was reflected by higher expression of MHC class II and costimulatory molecules (CD40, CD80, and CD86) and greater production of IL-12. On the contrary, BALB/c BMDCs secreted more IL-23, IL-6, IL-10, and TNF-α than C57BL/6 cells (Jiang et al., 2010). Overall, the data described above demonstrate that genetically defined differences in functionality between DCs from C57BL/6 and BALB/c mice may be phenotypically expressed during exposure to the microbial agent/infection, however, this reactivity also may depend on the nature of the agent and the host–agent immunobiology.

ECTV infection of GM-BM derived from C57BL/6 and BALB/c mice led to a profound repression of a set of chemokine and cytokine genes involved in Th1 [*Ccl3, Ccl4, Ccl5* (Lebre et al., 2005), *Cxcl2* (Seow et al., 2008), *Cxcl10* (Lebre et al., 2005), *Ifng, Il12a, Il12b*, and *Il16* (Lynch et al., 2003)] and Th2 [*Ccl2* (Liu et al., 2002), *Ccl8* (Lech and Anders, 2013), *Ccl11* (Dixon et al., 2006), *Ccl17* (Belperio et al., 2004), *Cxcl12* (Piao et al., 2012), *Mif* (Das et al., 2011), and *Tgfb1* (Maeda and Shiraishi, 1996)] immune response regulation. But, ECTV infection up-regulated mRNA transcript for IL-10 in GM-BM of both strains of mice. Despite the increase in gene expression, the level of IL-10 remained undetectable in the culture supernatants during 24 h incubation. We cannot exclude that GM-BM were cultured at too low cell density to detect IL-10 in our experimental model. On the other hand, it is possible that ECTV does not stimulate IL-10 production by GM-BM, similar to CPXV, which has been shown not to induce the secretion of IL-10 by different types of human DCs: monocyte-derived DCs, myeloid DCs, and plasmacytoid DCs (Hansen et al., 2011).

IL-10, first described as a Th2-polarizing cytokine, is an anti-inflammatory cytokine that suppresses pro-inflammatory response leading to increased pathogen dissemination and/or reduced pathology. IL-10 secreted by DCs may act in an autocrine manner to inhibit production of chemokines (such as CCL2, CCL5, CCL12, CXCL8, and CXCL10) and pro-inflammatory cytokines (such as IL-1α/β, IL-6, IL-12, IL-18, and TNF-α). Moreover, IL-10 may directly inhibit proliferation and production of IL-2, IFN-γ, IL-4, IL-5, and TNF-α by CD4^+^ T cells, thus it regulates both Th1 and Th2 immune responses (Couper et al., 2008). Additionally, IL-10 induces long-lasting T cell anergy and promotes the differentiation of naïve T cells into Tr1 cells in humans and mice (Gregori et al., 2010). The importance of cellular IL-10 in immune system regulation is supported by the fact that several viruses, including seven members of the *Poxviridae* family, encode orthologs of cellular IL-10, called viral IL-10s (vIL-10s), which have been acquired by viruses from their host during evolution (Ouyang et al., 2014).

It has been shown that CPXV induces *in vitro* secretion of IL-10 by BMDCs and RAW 264.7 macrophages at 24 hpi. Moreover, CPXV is able to induce higher IL-10 production *in vivo* than VACV (Spesock et al., 2011). Experiments with IL-10-deficient mice have indicated that after intranasal CPXV infection these mice exhibited similar weight loss and viral burdens as wild-type mice. However, IL-10-deficient mice were more susceptible to CPXV reinfection, because increased viral loads were observed in their lungs, what corresponded with lower antibody and CD8^+^ T cell responses compared to wild-type mice. The role of IL-10 during CPXV infection is probably beneficial for the virus, and IL-10 suppresses immunopathology in the lungs because IL-10-deficient mice after re-challenge with CPXV displayed greater bronchopneumonia than wild-type mice (Spesock et al., 2011). Meanwhile, using recombinant VACV expressing mouse IL-10 (mIL-10) it has been shown that in immunocompetent mice mIL-10 expressed from the VACV genome affected natural killer (NK) and virus-specific CTL activity, whereas in severe combined immunodeficient (SCID) mice VACV-mIL-10 infection resulted in increased NK cell activity and higher degree of virus clearance compared to infection with control VACV (Kurilla et al., 1993). In agreement with these studies, van Den Broek et al. (2000) have demonstrated that IL-10-deficient mice showed dramatically reduced viral titer in ovaries after intraperitoneal infection, suggesting a rapid viral clearance. Together, these data indicate that IL-10 is a potent cytokine in suppressing the immune response against VACV and may be a dominant factor for susceptibility to acute VACV infection (van Den Broek et al., 2000). On the hand, more recently it has been found, using multiphoton intravital microscopy imaging, that IL-10 produced locally in the skin of VACV-infected mice contributed to viral clearance, probably by shaping the innate immune response within the inflamed tissue and/or by reducing virus-induced inflammation (Cush et al., 2016). The up-regulation of IL-10 mRNA level observed in ECTV-infected GM-BM may therefore suggest that these cells possess a stronger ability to damp of the immune response and/or cross-regulate Th1 and Th2 immune responses. It is highly possible that IL-10 may be an important factor in the generation of non-protective Th2 immune response in susceptible BALB/c mice *in vivo*.

Our studies revealed that ECTV infection impairs inflammatory response and maturation of GM-BM in a strain-independent manner. At 24 hpi GM-BM from C57BL/6 and BALB/c mice displayed an altered production of several cytokines and chemokines with the exception of CCL3, which was produced at a higher concentration. Interestingly, mRNA transcript for CCL3 was down-regulated at this time point in ECTV-infected cells. It is known that mRNA expression does not always correlate with protein levels in mammalian cells (Vogel and Marcotte, 2012). Moreover, ECTV infection of GM-BM from both mouse strains down-regulated expression of MHC class I, and CD40 and CD80 co-stimulatory molecules, but increased the percentage of MHC II^+^ and CD86^+^ cells. Possibly, bystander non-infected GM-BM underwent partial maturation at the same time being the main source of CCL3. It has been demonstrated that highly attenuated modified vaccinia virus Ankara (MVA) induces phenotypic and functional maturation of bystander DCs resulting in production of a large array of cytokines and chemokines involved in T cell activation and recruitment, and regulation of inflammatory response, including CCL3 (Pascutti et al., 2011). However, ECTV encodes vCCI decoy receptor, EVM1, which is an abundantly secreted glycoprotein during infection and can bind the CC chemokines to form highly stable complexes with CCL3 and CCL5 (Arnold and Fremont, 2006). Our results also showed that the inhibition of inflammatory response and maturation of C57BL/6 and BALB/c GM-BM caused by ECTV was most pronounced after LPS treatment. This observation is in agreement with previous *in vitro* studies showing that other orthopoxviruses, such as VACV and CPXV severely affected maturation and activation of cDCs (Engelmayer et al., 1999; Jenne et al., 2000; Hansen et al., 2011).

As a master of immune inhibitory strategies, ECTV also altered the expression of chemokine receptors, such as CCR1, CCR5, and CCR7 on the C57BL/6 and BALB/c GM-BM surface, what may lead to their impaired migration (Jang et al., 2006). Humrich et al. (2007) have demonstrated that VACV infection targets chemokine-induced migration of DCs at multiple functional levels. VACV-infected mature DCs showed inability to migrate toward the lymphoid chemokines CCL19 and CXCL12 without apparent alterations in expression of surface chemokine receptors CXCR4 and CCR7. In fact, in VACV-infected immature or uninfected bystander DCs there is decreased or increased, respectively, expression of the inflammatory chemokine receptors CCR1 and CXCR1, which abrogates or intensifies their migration toward CCL3 and CCL5. Moreover, VACV-infected and uninfected bystander DCs are not able to up-regulate CCR7 expression after LPS treatment suggesting their disability to undergo chemokine receptor switch (Humrich et al., 2007). Additionally, poxviruses encode membrane cytokine and chemokine receptors to evade host immune responses (Felix and Savvides, 2017).

Taken together, our results indicate that *in vitro* ECTV infection of GM-BM, including cDCs, leads to their functional impairment independently of the genetic background of mice from which they were generated. ECTV-employed host-specific strategies to evade host antiviral immune response allow the virus to control GM-BM independently of the host resistance or susceptibility to severe mousepox. Moreover, our study confirms that ECTV is a master of immune inhibitory strategies showing a wide array of mechanisms for disrupting the innate and acquired immune functions of GM-BM. Better understanding of the virus interactions with cDCs, the most potent APCs, may help to elucidate additional mechanisms responsible for resistance or susceptibility to mousepox and that can lead to rational design means and ways of containing virus infection.

## Author Contributions

LS-D conceived and designed the study. LS-D, JC, ZN, and FT conducted real-time PCR experiments. LS-D, AW, and MG performed flow cytometry analysis. LS-D and JS performed ELISA. LS-D, JS, FT, and MG analyzed and interpreted the data. LS-D prepared figures and wrote the draft of the manuscript. All authors reviewed and approved the manuscript.

## Conflict of Interest Statement

The authors declare that the research was conducted in the absence of any commercial or financial relationships that could be construed as a potential conflict of interest. The reviewer RALR and handling Editor declared their shared affiliation.

## Supplementary Material

FIGURE S1 Characteristics of C57BL/6 and BALB/c GM-BM before and after MACS separation of CD11c^+^ cells. **(A)** Representative dot plot demonstrating gating strategy of isotype controls and CD11c and I-A/I-E staining. **(B)** Representative histograms demonstrating MFI of CD11c, CD11b, and CD205 expression on C57BL/6 and BALB/c GM-BM before and after MACS separation.

TABLE S1 Gene list description of Mouse Dendritic and Antigen Presenting Cell RT2 Profiler PCR Array (Qiagen).

## References

[B1] Arnold P. L. Fremont D. H. (2006). Structural determinants of chemokine binding by an ectromelia virus-encoded decoy receptor. *J. Virol.* 80 7439–7449. 10.1128/JVI.00576-06 16840324 PMC1563704

[B2] Belkaid Y. Piccirillo C. A. Mendez S. Shevach E. M. Sacks D. L. (2002). CD4+ CD25+ regulatory T cells control *Leishmania* major persistence and immunity. *Nature* 420 633–637. 10.1038/nature01152 12466842

[B3] Belperio J. A. Dy M. Murray L. Burdick M. D. Xue Y. Strieter R. M. (2004). The role of the Th2 CC chemokine ligand CCL17 in pulmonary fibrosis. *J. Immunol* 173 4692–4698. 10.4049/jimmunol.173.7.4692 15383605

[B4] Brownstein D. G. Gras L. (1995). Chromosome mapping of Rmp-4, a gonad-dependent gene encoding host resistance to mousepox. *J. Virol.* 69 6958–6964. 7474114 10.1128/jvi.69.11.6958-6964.1995 PMC189614

[B5] Chaudhri G. Panchanathan V. Buller R. M. L. van den Eertwegh A. J. M. Claassen E. Zhou J. (2004). Polarized type 1 cytokine response and cell-mediated immunity determine genetic resistance to mousepox. *Proc. Natl. Acad. Sci. U.S.A.* 101 9057–9062. 10.1073/pnas.0402949101 15184649 PMC428472

[B6] Couper K. N. Blount D. G. Riley E. M. (2008). IL-10: the master regulator of immunity to infection. *J. Immunol.* 180 5771–5777. 10.4049/jimmunol.180.9.5771 18424693

[B7] Cush S. S. Reynoso G. V. Kamenyeva O. Bennink J. R. Yewdell J. W. Hickman H. D. (2016). Locally produced IL-10 limits cutaneous *Vaccinia virus* spread. *PLOS Pathog.* 12:e1005493. 10.1371/journal.ppat.1005493 26991092 PMC4798720

[B8] Das R. Moss J. E. Robinson E. Roberts S. Levy R. Mizue Y. (2011). Role of macrophage migration inhibitory factor in the Th2 immune response to epicutaneous sensitization. *J. Clin. Immunol.* 31 666–680. 10.1007/s10875-011-9541-7 21559932 PMC3700537

[B9] Dixon H. Blanchard C. Deschoolmeester M. L. Yuill N. C. Christie J. W. Rothenberg M. E. (2006). The role of Th2 cytokines, chemokines and parasite products in eosinophil recruitment to the gastrointestinal mucosa during helminth infection. *Eur. J. Immunol.* 36 1753–1763. 10.1002/eji.200535492 16783848

[B10] Dolega P. Szulc-Dąbrowska L. Bossowska M. Mielcarska M. Nowak Z. Toka F. N. (2017). Innate immune gene transcript level associated with the infection of macrophages with ectromelia virus in two different mouse strains. *Viral. Immunol.* 5 315–329. 10.1089/vim.2016.0173 28453414

[B11] Engelmayer J. Larsson M. Subklewe M. Chahroudi A. Schmaljohn A. William C. (1999). *Vaccinia virus* inhibits the maturation of human dendritic cells: a novel mechanism of immune evasion. *J. Immunol.* 163 6762–6768. 10586075

[B12] Felix J. Savvides S. N. (2017). Mechanisms of immunomodulation by mammalian and viral decoy receptors: insights from structures. *Nat. Rev. Immunol.* 17 112–129. 10.1038/nri.2016.134 28028310

[B13] Filippi C. Hugues S. Cazareth J. Julia V. Glaichenhaus N. Ugolini S. (2003). CD4+ T cell polarization in mice is modulated by strain-specific major histocompatibility complex–independent differences within dendritic cells. *J. Exp. Med.* 198 201–209. 10.1084/jem.20021893 12860929 PMC2194066

[B14] Gregori S. Tomasoni D. Pacciani V. Scirpoli M. Battaglia M. (2010). Differentiation of type 1 T regulatory cells (Tr1) by tolerogenic DC-10 requires the IL-10-dependent ILT4/HLA-G pathway. *Blood* 116 935–944. 10.1182/blood-2009-07-234872 20448110

[B15] Hansen S. J. Rushton J. Dekonenko A. Chand H. S. Olson G. K. Hutt J. A. (2011). Cowpox virus inhibits human dendritic cell immune function by nonlethal, nonproductive infection. *Virology* 412 411–425. 10.1016/j.virol.2011.01.024 21334039 PMC3694803

[B16] Humrich J. Y. Thumann P. Greiner S. Humrich J. H. Averbeck M. Schwank C. (2007). *Vaccinia virus* impairs directional migration and chemokine receptor switch of human dendritic cells. *Eur. J. Immunol.* 37 954–965. 10.1002/eji.200636230 17357104

[B17] Jang M. H. Sougawa N. Tanaka T. Hirata T. Hiroi T. Tohya K. (2006). CCR7 is critically important for migration of dendritic cells in intestinal lamina propria to mesenteric lymph nodes. *J. Immunol.* 176 803–810. 10.4049/jimmunol.176.2.803 16393963

[B18] Jenne L. Hauser C. Arrighi J. F. Saurat J. H. Hugin A. W. (2000). Poxvirus as a vector to transduce human dendritic cells for immunotherapy: abortive infection but reduced APC function. *Gene Ther.* 7 1575–1583. 11021596 10.1038/sj.gt.3301287

[B19] Jiang X. Shen C. Yu H. Karunakaran K. P. Brunham R. C. (2010). Differences in innate immune responses correlate with differences in murine susceptibility to *Chlamydia muridarum* pulmonary infection. *Immunology* 129 556–566. 10.1111/j.1365-2567.2009.03157.x 20102413 PMC2842502

[B20] Kaiko E. G. Horvat J. C. Beagley K. W. Hansbro P. M. (2008). Immunological decision-making: how does the immune system decide to mount a helper T-cell response? *Immunology* 123 326–338. 10.1111/j.1365-2567.2007.02719.x 17983439 PMC2433332

[B21] Koike E. Takano H. Inoue K. Yanagisawa R. (2008). Accelerated differentiation of bone marrow-derived dendritic cells. *Int. Immunopharmacol.* 8 1737–1743. 10.1016/j.intimp.2008.08.006 18775800

[B22] Kurilla M. G. Swaminathan S. Welsh R. M. Kieff E. Brutkiewicz R. R. (1993). Effects of virally expressed interleukin-10 on *Vaccinia virus* infection in mice. *J. Virol.* 67 7623–7628. 8230481 10.1128/jvi.67.12.7623-7628.1993 PMC238230

[B23] Le Nouën C. Hillyer P. Winter C. C. McCarty T. Rabin R. L. Collins P. L. (2011). Low CCR7-mediated migration of human monocyte derived dendritic cells in response to human respiratory syncytial virus and human metapneumovirus. *PLOS Pathog.* 7:e1002105. 10.1371/journal.ppat.1002105 21731495 PMC3121884

[B24] Lebre M. C. Burwell T. Vieira P. L. Lora J. Coyle A. J. Kapsenberg M. L. (2005). Differential expression of inflammatory chemokines by Th1- and Th2-cell promoting dendritic cells: a role for different mature dendritic cell populations in attracting appropriate effector cells to peripheral sites of inflammation. *Immunol. Cell Biol.* 83 525–535. 10.1111/j.1440-1711.2005.01365.x 16174103

[B25] Lech M. Anders H.-J. (2013). Macrophages and fibrosis: how resident and infiltrating mononuclear phagocytes orchestrate all phases of tissue injury and repair. *Biochim. Biophys. Acta* 1832 989–997. 10.1016/j.bbadis.2012.12.001 23246690

[B26] Liu T. Matsuguchi T. Tsuboi N. Yajima T. Yoshikai Y. (2002). Differences in expression of Toll-like receptors and their reactivities in dendritic cells in BALB/c and C57BL/6 mice. *Infect. Immun.* 70 6638–6645. 10.1128/IAI.70.12.6638-6645.2002 12438336 PMC133006

[B27] Liu T. Nishimura H. Matsuguchi T. Yoshikai Y. (2000). Difference in interleukin-12 and -15 production by dendritic cells at the early stage of *Listeria monocytogenes* infection between BALB/c and C57BL/6 mice. *Cell. Immunol.* 202 31–40. 10.1128/IAI.70.12.6638-6645.2002 10873304

[B28] Lutz M. B. (2016). Induction of CD4+ regulatory and polarized effector/helper T cells by dendritic cells. *Immune Netw.* 16 13–25. 10.4110/in.2016.16.1.13 26937228 PMC4770096

[B29] Lutz M. B. Kukutsch N. Ogilvie A. L. Rössner S. Koch F. Romani N. (1999). An advanced culture method for generating large quantities of highly pure dendritic cells from mouse bone marrow. *J. Immunol. Methods* 223 77–92. 10.1016/S0022-1759(98)00204-X 10037236

[B30] Lynch E. A. Heijens C. A. Horst N. F. Center D. M. Cruikshank W. W. (2003). Cutting edge: IL-16/CD4 preferentially induces Th1 cell migration: requirement of CCR5. *J. Immunol.* 171 4965–4968. 10.4049/jimmunol.171.10.4965 14607889

[B31] Maeda H. Shiraishi A. (1996). TGF-beta contributes to the shift toward Th2-type responses through direct and IL-10-mediated pathways in tumor-bearing mice. *J. Immunol.* 156 73–78. 8598496

[B32] McCollum A. M. Damon I. K. (2014). Human monkeypox. *Clin. Infect. Dis.* 58 260–267. 10.1093/cid/cit703 24158414 PMC5895105

[B33] Mehta V. B. Hart J. Wewers D. (2000). ATP stimulated release of IL-1β and IL-18 requires priming by LPS and is independent of caspase-1 cleavage. *J. Biol. Chem.* 276 3820–3826. 10.1074/jbc.M006814200 11056157

[B34] Niedbala W. Wei X. Q. Piedrafita D. Xu D. Liew F. Y. (1999). Effects of nitric oxide on the induction and differentiation of Th1 cells. *Eur. J. Immunol.* 29 2498–2505. 10458764 10.1002/(SICI)1521-4141(199908)29:08<2498::AID-IMMU2498>3.0.CO;2-M

[B35] Ouyang P. Rakus K. van Beurden S. J. Westphal A. H. Davison A. J. Gatherer D. (2014). IL-10 encoded by viruses: a remarkable example of independent acquisition of a cellular gene by viruses and its subsequent evolution in the viral genome. *J. Gen. Virol.* 95 245–262. 10.1099/vir.0.058966-0 24225498

[B36] Palumbo M. L. Canzobre M. C. Pascuan C. G. Ríos H. Wald M. Genaro A. M. (2010). Stress induced cognitive deficit is differentially modulated in BALB/c and C57Bl/6 mice: correlation with Th1/Th2 balance after stress exposure. *J. Neuroimmunol.* 218 12–20. 10.1016/j.jneuroim.2009.11.005 19942299

[B37] Parker S. Siddiqui A. M. Painter G. Schriewer J. Buller R. M. (2010). Ectromelia virus infections of mice as a model to support the licensure of anti-orthopoxvirus therapeutics. *Viruses* 2 1918–1932. 10.3390/v2091918 21994714 PMC3185751

[B38] Pasare C. Medzhitov R. (2003). Toll pathway-dependent blockade of CD4+ CD25+ T cell-mediated suppression by dendritic cells. *Science* 299 1033–1036. 10.1126/science.1078231 12532024

[B39] Pascutti M. F. Rodriguez A. M. Falivene J. Giavedoni L. Drexler I. Gherardi M. M. (2011). Interplay between modified *Vaccinia virus* Ankara and dendritic cells: phenotypic and functional maturation of bystander dendritic cells. *J. Virol.* 85 5532–5545. 10.1128/JVI.02267-10 21411535 PMC3094969

[B40] Pejawar S. S. Parks G. D. Alexander-Miller M. A. (2005). Abortive versus productive viral infection of dendritic cells with a paramyxovirus results in differential upregulation of select costimulatory molecules. *J. Virol.* 79 7544–7557. 10.1128/JVI.79.12.7544-7557.2005 15919909 PMC1143689

[B41] Piao H.-L. Tao Y. Zhu R. Wang S.-C. Tang C. L. Fu Q. (2012). The CXCL12/CXCR4 axis is involved in the maintenance of Th2 bias at the maternal/fetal interface in early human pregnancy. *Cell. Mol. Immunol.* 9 423–430. 10.1038/cmi.2012.23 22885527 PMC4002329

[B42] Riedel S. (2005). Smallpox and biological warfare: a disease revisited. *Proc. (Bayl. Univ. Med. Cent.)* 18 13–20. 16200143 10.1080/08998280.2005.11928026 PMC1200695

[B43] Russe O. Q. Möser C. V. Kynast K. L. King T. S. Olbrich K. Grösch S. (2014). LPS inhibits caspase 3-dependent apoptosis in RAW264.7 macrophages induced by the AMPK activator AICAR. *Biochem. Biophys. Res. Commun.* 447 520–525. 10.1016/j.bbrc.2014.04.008 24732361

[B44] Sacks D. Noben-Trauth N. (2002). The immunology of susceptibility and resistance to *Leishmania* major in mice. *Nat. Rev. Immunol.* 2 845–858. 10.1038/nri933 12415308

[B45] Sei J. J. Haskett S. Kaminsky L. W. Lin E. Truckenmiller M. E. Bellone C. J. (2015). Peptide-MHC-I from endogenous antigen outnumber those from exogenous antigen, irrespective of APC phenotype or activation. *PLOS Pathog.* 11:e1004941. 10.1371/journal.ppat.1004941 26107264 PMC4479883

[B46] Seow S. W. Rahmat J. N. Bay B. H. Lee Y. K. Mahendran R. (2008). Expression of chemokine/cytokine genes and immune cell recruitment following the instillation of *Mycobacterium bovis, Bacillus* Calmette–Guérin or *Lactobacillus rhamnosus* strain GG in the healthy murine bladder. *Immunology* 124 419–427. 10.1111/j.1365-2567.2007.02792.x 18217952 PMC2440836

[B47] Spesock A. H. Barefoot B. E. Ray C. A. Kenan D. J. Gunn M. D. Ramsburg E. A. (2011). Cowpox virus induces interleukin-10 both *in vitro* and in vivo. *Virology* 417 87–97. 10.1016/j.virol.2011.05.010 21658738 PMC3212434

[B48] Stanford M. M. McFadden G. Karupiah G. Chaudhri G. (2007). Immunopathogenesis of poxvirus infections: forecasting the impending storm. *Immunol. Cell Biol.* 85 93–102. 10.1038/sj.icb.7100033 17228320

[B49] Szulc-Dabrowska L. Gregorczyk K. P. Struzik J. Boratynska-Jasinska A. Szczepanowska J. Wyzewski Z. (2016). Remodeling of the fibroblast cytoskeletal architecture during the replication cycle of *Ectromelia virus*: a morphological *in vitro* study in a murine cell line. *Cytoskeleton* 7 396–417. 10.1002/cm.21308 27169394

[B50] Szulc-Dąbrowska L. Struzik J. Ostrowska A. Guzera M. Toka F. N. Bossowska-Nowicka M. (2017). Functional paralysis of GM-CSF–derived bone marrow cells productively infected with *Ectromelia virus*. *PLOS ONE* 12:e0179166. 10.1371/journal.pone.0179166 28604814 PMC5467855

[B51] Trunova G. V. Makarova O. V. Diatroptov M. E. Bogdanova I. M. Mikchailova L. P. Abdulaeva S. O. (2011). Morphofunctional characteristic of the immune system in BALB/c and C57Bl/6 mice. *Bull. Exp. Biol. Med.* 151 99–102. 22442812 10.1007/s10517-011-1268-1

[B52] van Den Broek M. Bachmann M. F. Köhler G. Barner M. Escher R. Zinkernagel R. (2000). IL-4 and IL-10 antagonize IL-12-mediated protection against acute *Vaccinia virus* infection with a limited role of IFN-gamma and nitric oxide synthetase 2. *J. Immunol.* 164 371–378. 10.4049/jimmunol.164.1.371 10605032

[B53] Vogel C. Marcotte E. M. (2012). Insights into the regulation of protein abundance from proteomic and transcriptomic analyses. *Nat. Rev. Genet.* 13 227–232. 10.1038/nrg3185 22411467 PMC3654667

[B54] Watanabe K. Numata K. Ito T. Takagi K. Matsukawa A. (2004). Innate immune response in Th1- and Th2-dominant mouse strains. *Shock* 22 460–466. 10.1097/01.shk.0000142249.08135.e9 15489639

[B55] Zhang W. Chen Z. Li F. Kamencic H. Juurlink B. Gordon J. R. (2003). Tumour necrosis factor-α (TNF-α) transgene-expressing dendritic cells (DCs) undergo augmented cellular maturation and induce more robust T-cell activation and anti-tumour immunity than DCs generated in recombinant TNF-α. *Immunology* 108 177–188. 10.1046/j.1365-2567.2003.01489.x 12562326 PMC1782887

