# Supplementary figures and images for: The in Vitro Inhibitory Effect of Ectromelia Virus Infection on Innate and Adaptive Immune Properties of GM-CSF-Derived Bone Marrow Cells Is Mouse Strain-Independent
Source: Front Microbiol. 2017 Dec 19;8:2539. doi: 10.3389/fmicb.2017.02539 (PMC5742134; doi:10.3389/fmicb.2017.02539)

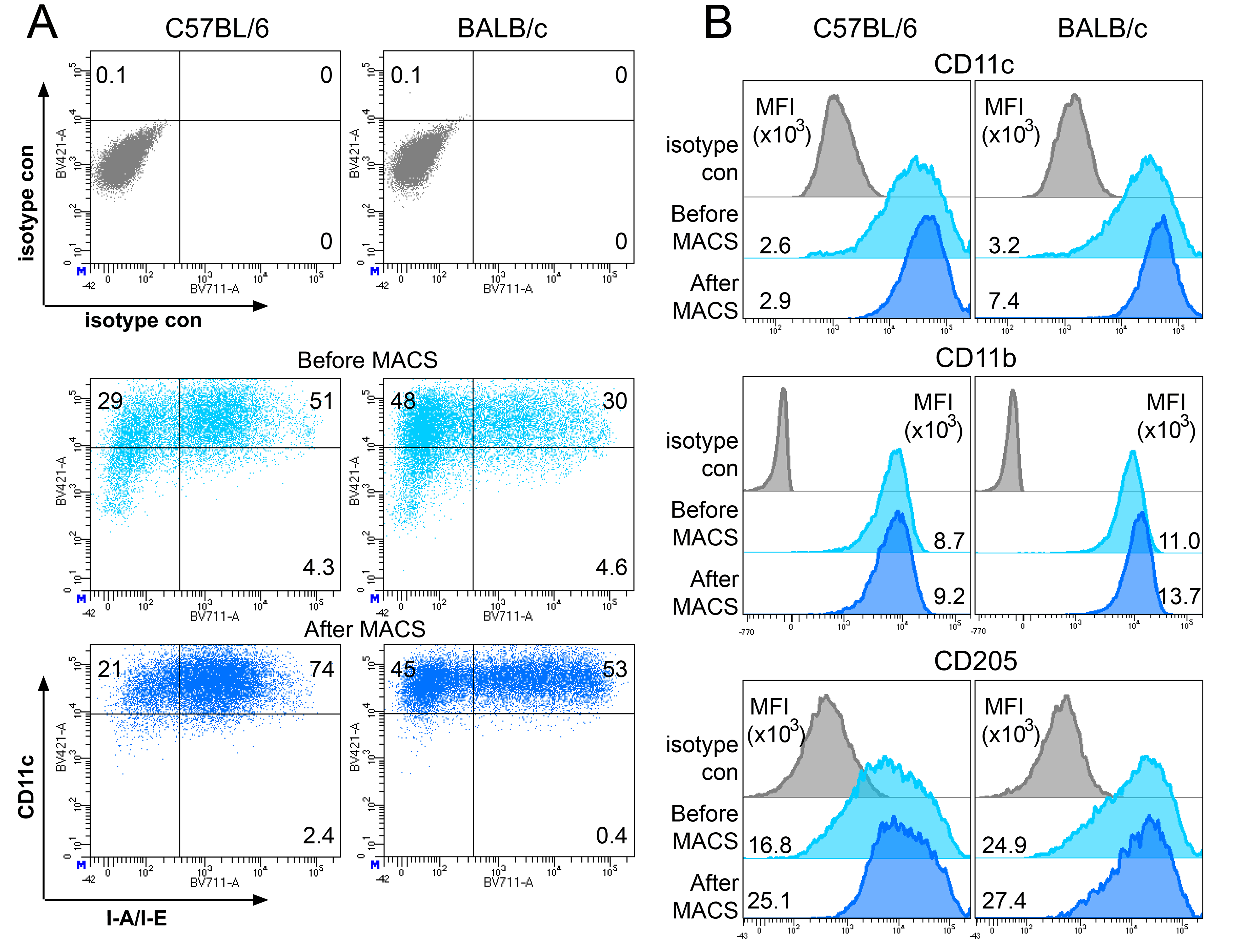

Supplement: FIGURE S1 — Characteristics of C57BL/6 and BALB/c GM-BM before and after MACS separation of CD11c+ cells. (A) Representative dot plot demonstrating gating strategy of isotype controls and CD11c and I-A/I-E staining. (B) Representative histograms demonstrating MFI of CD11c, CD11b, and CD205 expression on C57BL/6 and BALB/c GM-BM before and after MACS separation. [file Image_1.TIF]
