# Supplementary material for: The in Vitro Inhibitory Effect of Ectromelia Virus Infection on Innate and Adaptive Immune Properties of GM-CSF-Derived Bone Marrow Cells Is Mouse Strain-Independent
Source: Front Microbiol. 2017 Dec 19;8:2539. doi: 10.3389/fmicb.2017.02539 (PMC5742134; doi:10.3389/fmicb.2017.02539)
Supplement: TABLE S1 — Gene list description of Mouse Dendritic and Antigen Presenting Cell RT2 Profiler PCR Array (Qiagen). [file Table_1.PDF]

Table S1 Gene list description of Mouse Dendritic and Antigen Presenting Cell RT^2 Profiler PCR Array (Qiagen)

Position	Symbol	Description
A01	*Ccl11*	Chemokine (C-C motif) ligand 11
A02	*Ccl12*	Chemokine (C-C motif) ligand 12
A03	*Ccl17*	Chemokine (C-C motif) ligand 17
A04	*Ccl19*	Chemokine (C-C motif) ligand 19
A05	*Ccl2*	Chemokine (C-C motif) ligand 2
A06	*Ccl20*	Chemokine (C-C motif) ligand 20
A07	*Ccl3*	Chemokine (C-C motif) ligand 3
A08	*Ccl4*	Chemokine (C-C motif) ligand 4
A09	*Ccl5*	Chemokine (C-C motif) ligand 5
A10	*Ccl7*	Chemokine (C-C motif) ligand 7
A11	*Ccl8*	Chemokine (C-C motif) ligand 8
A12	*Ccr1*	Chemokine (C-C motif) receptor 1
B01	*Ccr2*	Chemokine (C-C motif) receptor 2
B02	*Ccr3*	Chemokine (C-C motif) receptor 3
B03	*Ccr5*	Chemokine (C-C motif) receptor 5
B04	*Ccr9*	Chemokine (C-C motif) receptor 9
B05	*Cd1d1*	CD1d1 antigen
B06	*Cd1d2*	CD1d2 antigen
B07	*Cd2*	CD2 antigen
B08	*Cd209a*	CD209a antigen
B09	*Cd28*	CD28 antigen
B10	*Cd33*	CD33 antigen
B11	*Cd36*	CD36 antigen
B12	*Cd4*	CD4 antigen
C01	*Cd40*	CD40 antigen
C02	*Cd40lg*	CD40 ligand
C03	*Cd44*	CD44 antigen
C04	*Cd74*	CD74 antigen
C05	*Cd80*	CD80 antigen
C06	*Cd86*	CD86 antigen
C07	*Cd8a*	CD8 antigen, alpha chain
C08	*Cdc42*	Cell division cycle 42 homolog (S. cerevisiae)
C09	*Cdkn1a*	Cyclin-dependent kinase inhibitor 1A (P21)

C10	*Cebpa*	CCAAT/enhancer binding protein (C/EBP), alpha
C11	*Clec4b2*	C-type lectin domain family 4, member b2
C12	*Csf1r*	Colony stimulating factor 1 receptor
D01	*Csf2*	Colony stimulating factor 2 (granulocyte-macrophage)
D02	*Cxcl1*	Chemokine (C-X-C motif) ligand 1
D03	*Cxcl10*	Chemokine (C-X-C motif) ligand 10
D04	*Cxcl12*	Chemokine (C-X-C motif) ligand 12
D05	*Cxcl2*	Chemokine (C-X-C motif) ligand 2
D06	*Cxcr1*	Chemokine (C-X-C motif) receptor 1
D07	*Cxcr4*	Chemokine (C-X-C motif) receptor 4
D08	*Erbb2*	V-erb-b2 erythroblastic leukemia viral oncogene homolog 2
D09	*Fas*	Fas (TNF receptor superfamily member 6)
D10	*Fcer1a*	Fc receptor, IgE, high affinity I, alpha polypeptide
D11	*Fcer2a*	Fc receptor, IgE, low affinity II, alpha polypeptide
D12	*Fcgr1*	Fc receptor, IgG, high affinity I
E01	*Fcgrt*	Fc receptor, IgG, alpha chain transporter
E02	*Flt3*	FMS-like tyrosine kinase 3
E03	*Flt3l*	FMS-like tyrosine kinase 3 ligand
E04	*H2-DMa*	Histocompatibility 2, class II, locus DMa
E05	*Icam1*	Intercellular adhesion molecule 1
E06	*Icam2*	Intercellular adhesion molecule 2
E07	*Ifng*	Interferon gamma
E08	*Il10*	Interleukin 10
E09	*Il12a*	Interleukin 12A
E10	*Il12b*	Interleukin 12b
E11	*Il16*	Interleukin 16
E12	*Il2*	Interleukin 2
F01	*Il6*	Interleukin 6
F02	*Irf7*	Interferon regulatory factor 7
F03	*Itgam*	Integrin alpha M
F04	*Itgb2*	Integrin beta 2
F05	*Lrp1*	Low density lipoprotein receptor-related protein 1
F06	*Lyn*	Yamaguchi sarcoma viral (v-yes-1) oncogene homolog
F07	*Mif*	Macrophage migration inhibitory factor
F08	*Nfkb1*	Nuclear factor of kappa light polypeptide gene enhancer in B-cells 1, p105
F09	*Ptprc*	Protein tyrosine phosphatase, receptor type, C

F10	*Rac1*	RAS-related C3 botulinum substrate 1
F11	*Rag1*	Recombination activating gene 1
F12	*Rela*	V-rel reticuloendotheliosis viral oncogene homolog A (avian)
G01	*Relb*	Avian reticuloendotheliosis viral (v-rel) oncogene related B
G02	*Stat3*	Signal transducer and activator of transcription 3
G03	*Tap2*	Transporter 2, ATP-binding cassette, sub-family B (MDR/TAP)
G04	*Tapbp*	TAP binding protein
G05	*Tgfb1*	Transforming growth factor, beta 1
G06	*Thbs1*	Thrombospondin 1
G07	*Tlr1*	Toll-like receptor 1
G08	*Tlr2*	Toll-like receptor 2
G09	*Tlr7*	Toll-like receptor 7
G10	*Tlr9*	Toll-like receptor 9
G11	*Tnf*	Tumor necrosis factor
G12	*Tnfsf11*	Tumor necrosis factor (ligand) superfamily, member 11
H01	*Actb*	Actin, beta
H02	*B2m*	Beta-2 microglobulin
H03	*Gapdh*	Glyceraldehyde-3-phosphate dehydrogenase
H04	*Gusb*	Glucuronidase, beta
H05	*Hsp90ab1*	Heat shock protein 90 alpha (cytosolic), class B member 1
H06	MGDC	Mouse Genomic DNA Contamination
H07	RTC	Reverse Transcription Control
H08	RTC	Reverse Transcription Control
H09	RTC	Reverse Transcription Control
H10	PPC	Positive PCR Control
H11	PPC	Positive PCR Control
H12	PPC	Positive PCR Control
